# TDMA-Based LoRa IoT Architecture with FreeRTOS for Real-Time Multi-Node Bridge Structural Health Monitoring

**DOI:** 10.3390/s26144381

**Published:** 2026-07-10

**Authors:** Thanh Binh Ngo, Quang Huy Le, Ngoc Quy Vu, Xuan Chieu Luong, Quang Binh Pham, Timothy Roberts, Andy Nguyen

**Affiliations:** 1 Department of Electrical and Electronic Engineering, University of Transport and Communications, Hanoi 100000, Vietnam; ngobinh74@utc.edu.vn; 2 FPT Software, Hanoi 100000, Vietnam; huylq61@fpt.com; 3 Center for Transport Science and Technology, University of Transport and Communications, Hanoi 100000, Vietnam; quygtvt2014@utc.edu.vn; 4 Department of Highway and Traffic Engineering, University of Transport and Communications, Hanoi 100000, Vietnam; 5 Dat Phuong Group Joint Stock Company, Hanoi 100000, Vietnam; binhpq@datphuong.vn; 6 School of Science, Engineering and Digital Technologies, University of Southern Queensland, Springfield, QLD 4300, Australia; timothy.roberts@unisq.edu.au

**Keywords:** IoT, RTOS, TDMA, smart sensing systems, structural health monitoring

## Abstract

**Highlights:**

**What are the main findings?**

- The proposed TDMA-based LoRa + FreeRTOS architecture enables predictable, bounded-latency multi-node SHM communication, achieving microsecond-level timing jitter (<15μs) and keeping total processing overhead <10 ms in the evaluated three-node deployment.
- System-level evaluation confirms >95% packet delivery ratio and stable ≈400 ms end-to-end latency across the complete sensing–uplink–gateway–backend pipeline, validating reliable real-time operation in practical bridge monitoring scenarios.

**What is the implications of the main finding?**

- The results indicate that TDMA communication and RTOS-driven task scheduling support reliable multi-node SHM operation in the tested three-node deployment. Larger-scale operation is treated as an architectural scalability potential supported by TDMA capacity analysis rather than as an experimentally proven maximum network size.

**Abstract:**

Structural health monitoring (SHM) systems based on Internet of Things (IoT) technologies have become an effective approach for continuous monitoring of bridge infrastructures. However, many wireless monitoring systems relying on LoRaWAN or contention-based communication suffer from packet collisions, unpredictable latency, and limited scalability when multiple sensing nodes operate simultaneously. To address these limitations, this study proposes a soft real-time LoRa-based IoT architecture for bridge SHM using a time division multiple access (TDMA) communication framework implemented on an embedded real-time platform. The proposed system integrates distributed vibration sensing nodes, a TDMA-enabled LoRa communication layer, an ESP32-based gateway, and a web-based monitoring database for remote visualization and analysis. The architecture leverages FreeRTOS (v10.4.3) for system-level task scheduling, enabling concurrent execution of sensing, communication, and networking processes across the dual-core ESP32-WROOM-32D platform. Experimental results obtained using a laboratory-scale cable-stayed bridge model demonstrate stable multi-node communication with a packet delivery ratio exceeding 95% and predictable TDMA-scheduled transmission cycles with TDMA slots of 100–200 ms under the evaluated operating conditions. The experiments validate end-to-end operation using a representative three-node deployment, while broader scalability is evaluated analytically through the TDMA capacity model and identified as future work for larger physical deployments.

## 1. Introduction

Bridges are critical components of transportation infrastructure and their safety and reliability are essential for economic activity and public safety. Aging structures, increasing traffic loads, and environmental effects have made continuous monitoring of bridge conditions increasingly important. Structural Health Monitoring (SHM) systems aim to provide timely information on structural behavior by collecting and analyzing vibration and response data from distributed sensors deployed in civil infrastructure such as bridges [1,2]. Recent advances in the Internet of Things (IoT) technologies have enabled low-cost and scalable structural health monitoring systems that can continuously collect and transmit sensor data from distributed nodes [3,4,5]. In such systems, multiple sensor nodes equipped with inertial measurement units (IMUs) are deployed along the structure to capture vibration data, which are then transmitted to a central gateway for storage, visualization, and analysis.

Among LPWAN technologies, LoRa has emerged as an attractive wireless communication technology for IoT monitoring applications due to its long communication range, low power consumption, and robustness in harsh environments [6,7,8]. However, bridge SHM systems often require predictable communication behavior, bounded latency, and reliable data delivery, especially as the number of sensor nodes increases. Conventional LoRa-based systems typically rely on contention-based channel access or LoRaWAN protocols, which may lead to packet collisions, unpredictable latency, and reduced communication performance in multi-node deployments. These limitations pose challenges for time-sensitive SHM applications that require synchronized data acquisition and bounded-latency communication, especially when multiple sensor nodes transmit periodically over a shared wireless channel [9].

To address these challenges, Time Division Multiple Access (TDMA) has been widely recognized as an effective medium access control strategy to minimize packet collisions and provide bounded delay. Asynchronous and synchronous TDMA variants have been shown to significantly reduce latency and energy consumption in LoRa IoT networks [10]. By assigning dedicated transmission slots to sensor nodes, TDMA enables scheduled uplink access and improves channel utilization in multi-node scenarios. However, implementing TDMA over LoRa requires careful consideration of time synchronization, radio time-on-air (ToA), guard intervals, real-time task scheduling, and stochastic wireless-channel effects on resource-constrained embedded platforms. Several LoRa-based SHM systems have been reported in the literature [3,11,12,13]. These works demonstrate the practicality of LoRa for long-range structural monitoring. However, TDMA-based predictable communication has not been widely explored in this context. In particular, existing systems often rely on LoRaWAN or contention-based access, showing that contention-based LoRaWAN/ALOHA schemes experience high collision rates and unpredictable latency when used in multi-node deployments, making them unsuitable for bounded-latency SHM applications [14].

FreeRTOS is one of the most widely adopted real-time operating systems for embedded IoT platforms due to its lightweight architecture, portability, and efficient task management mechanisms. By using FreeRTOS, sensing, communication, and networking functions can be implemented as independent tasks with controlled execution priorities. This capability is particularly beneficial for multi-node wireless communication systems where precise timing coordination is required for deterministic TDMA scheduling.

Despite these advancements, existing LoRa-based SHM systems remain limited in terms of communication determinism and system-level integration [12,15,16,17,18]. Many implementations still rely on contention-based communication protocols, which may lead to packet collisions and unpredictable transmission latency when multiple nodes attempt to transmit simultaneously [6,19]. Furthermore, prior works often address individual components—such as sensing hardware, wireless communication, or cloud-based monitoring—without jointly considering synchronized sensing, bounded-latency communication, and soft real-time embedded execution within a unified system architecture. This limitation restricts their applicability in time-sensitive SHM scenarios requiring predictable multi-node coordination.

In this paper, we present the design and implementation of an end-to-end SHM-IoT architecture for bridge monitoring based on a TDMA-enabled LoRa communication scheme. The proposed system integrates IMU-based sensor nodes, software-based time synchronization using periodic LoRa beacons, a FreeRTOS-based real-time processing pipeline, an ESP32 gateway, MQTT data streaming through a Mosquitto broker (v2.0.18), a MongoDB (v7.0.5) database, and a React (v18.2.0)-based web dashboard.

To address the communication reliability and multi-node coordination challenges in LoRa-based SHM systems, the proposed architecture incorporates PHY (physical layer)-aware TDMA slot design based on LoRa Time-on-Air analysis, enabling bounded-latency transmission scheduling under practical radio configurations. In addition, software-based synchronization mechanisms and RTOS-driven task management are employed to ensure predictable sensing and communication behavior on resource-constrained embedded platforms.

The primary objective of this work is to validate the feasibility and end-to-end integration of the proposed SHM-IoT architecture rather than to experimentally determine the maximum achievable network scale. Therefore, the experimental study focuses on a representative multi-node deployment, while broader scalability considerations are analyzed through the TDMA capacity model and discussed as future work. The system is implemented and experimentally validated using a laboratory-scale cable-stayed bridge model, where vibration data from multiple sensor nodes are collected and visualized in real time. Furthermore, quantitative evaluations are conducted to assess communication reliability and system performance, including packet delivery ratio (PDR), end-to-end latency, throughput, and node power consumption.

The main contributions of this work are summarized as follows:

- A deterministic LoRa communication architecture based on TDMA scheduling designed specifically for multi-node bridge SHM applications.
- A system-level embedded implementation using FreeRTOS on the ESP32-WROOM-32D platform that enables concurrent management of sensing, communication, and networking tasks.
- A complete end-to-end IoT monitoring framework integrating distributed sensing nodes, LoRa gateway communication, MQTT data streaming, and a web-based monitoring dashboard.
- Experimental validation of the proposed system demonstrating reliable multi-node communication with a packet delivery ratio exceeding 95% and predictable transmission cycles with TDMA slots of approximately 100–200 ms.

The remainder of this paper is organized as follows. Section 2 describes the overall system architecture of the proposed IoT-based bridge monitoring system. Section 3 presents the design of the TDMA communication mechanism and the embedded real-time implementation. Section 4 reports experimental results obtained from the multi-node deployment. Section 5 discusses the system performance and practical implications for SHM applications. Finally, Section 6 concludes the paper and outlines future research directions.

## 2. Related Work

Structural Health Monitoring (SHM) has become an important technique for assessing the safety and operational condition of large civil infrastructure such as bridges. Traditional SHM systems typically relied on wired sensing networks and centralized data acquisition units, which require complex installation and high maintenance costs, limiting scalability for large deployments. With the emergence of wireless sensor networks (WSNs), distributed sensing nodes can be deployed more flexibly to collect structural response measurements and transmit the data wirelessly to a central gateway for analysis [1,20].

Recent research has further integrated Internet of Things (IoT) technologies into SHM systems to enable remote monitoring, automated data collection, and long-term infrastructure management. IoT-based monitoring platforms allow sensor nodes to transmit measurements to cloud services where data can be stored, processed, and visualized through web-based dashboards. Several studies have demonstrated the feasibility of such architectures for vibration monitoring and infrastructure condition assessment [3,5]. These systems provide a cost-effective approach for continuous monitoring of civil structures.

Among the available wireless communication technologies for SHM applications, Low Power Wide Area Network (LPWAN) solutions such as LoRa and LoRaWAN have gained significant attention due to their long communication range and low power consumption. Compared with short-range technologies such as ZigBee (IEEE 802.15.4) [21], LoRa-based communication can provide kilometer-scale coverage, which is particularly suitable for monitoring large infrastructure such as bridges and transportation systems. Several studies have investigated LoRa-based sensing architectures for infrastructure monitoring, including bridge monitoring and construction site monitoring [12,13,15].

Despite these advantages, existing LoRaWAN-based systems commonly rely on an ALOHA-based medium access mechanism. This random access strategy may lead to packet collisions and unpredictable communication latency when multiple sensor nodes attempt to transmit simultaneously [6]. Such limitations are critical for SHM applications, where synchronized measurements and reliable data delivery are often required for accurate structural analysis.

To mitigate these issues, recent works have explored synchronized sensing and coordinated transmission mechanisms for LoRa-based monitoring systems [16]. These approaches improve measurement alignment across sensor nodes and reduce transmission conflicts. However, most existing implementations focus on individual subsystems, such as sensing hardware or wireless communication modules. Relatively limited work addresses a complete system architecture that jointly considers PHY-aware communication scheduling, real-time embedded firmware execution, and scalable IoT-based data management for SHM applications.

In traditional wireless sensor networks (WSNs), beacon-driven TDMA protocols based on location signals are commonly used to determine superframe structure and coordinate communication in time slots [9]. In the context of LoRa-based structural health monitoring (SHM) systems, synchronization techniques have been used to support time-synchronized sensing and structural vibration analysis [16]. Furthermore, recent studies on real-time LoRa communication highlight the need for coordinated transmission scheduling between nodes to ensure predictable latency and reliable data transmission delivery [10,14].

To position the proposed system within the existing literature, Table 1 compares representative SHM networking solutions in terms of communication protocol, real-time execution support, synchronization mechanism, communication latency characteristics, and network scalability and is consistent with findings from existing SHM monitoring architectures.

Multiple LoRa-based SHM deployments rely on LoRaWAN’s ALOHA access, which has been experimentally shown to suffer from high collision probability and non-deterministic latency in multi-node networks [14,22]. Additionally, widely used LoRa SHM systems typically emphasize sensing hardware and long-range connectivity, without incorporating TDMA-based scheduled access or real-time embedded execution [11,15].

To address these challenges, this paper proposes a TDMA-enabled LoRa SHM architecture that integrates PHY-aware slot design, software-based time synchronization, FreeRTOS-driven real-time task scheduling, and a scalable IoT data pipeline for bridge monitoring applications.

## 3. System Architecture

### 3.1. System Overview

**Figure 1:**
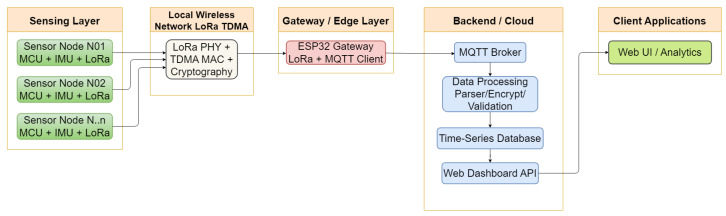
System architecture. Distributed IMU sensor nodes communicate with an ESP32 gateway through a TDMA-based LoRa network. The gateway aggregates uplink data and forwards them via MQTT to a cloud backend for processing, storage, and real-time visualization.

The proposed system is an end-to-end SHM architecture designed to enable periodic, synchronized, and predictable multi-node vibration data acquisition for bridge monitoring applications. Similar IoT-based SHM frameworks have been investigated in recent studies focusing on low-power sensing systems and distributed monitoring architectures [3,4,5]. Unlike the existing systems, the proposed architecture explicitly integrates PHY-aware TDMA communication, RTOS-based firmware execution, and an end-to-end IoT data pipeline to achieve predictable multi-node vibration data collection. As illustrated in Figure 1, the system consists of three main layers: distributed sensor nodes deployed on the bridge structure, a central LoRa gateway, and a backend platform including data transmission, storage, and visualization services.

At the sensing layer, multiple low-power sensor nodes equipped with inertial measurement units (IMUs) are installed at selected locations on the bridge to capture vibration signals. Each sensor node operates independently and periodically samples IMU data at a fixed interval. Such sensing configurations are commonly used in wireless SHM systems to monitor modal characteristics and structural responses [3,11,16]. To support multi-node operation and avoid wireless collisions, the system adopts a time-slotted communication scheme in which each sensor node is assigned a dedicated transmission window within a repeating communication frame.

The communication layer is based on a star topology, where all sensor nodes communicate directly with a central gateway using LoRa links. Instead of employing a contention-based access mechanism, the gateway periodically broadcasts a synchronization beacon that defines the start of each TDMA frame. Upon receiving the beacon, sensor nodes align their local clocks and determine their assigned transmission slots within the frame. This software-based synchronization mechanism enables coordinated data transmission while maintaining compatibility with standard LoRa transceivers.

At the gateway layer, an ESP32-based device acts as the LoRa concentrator and system coordinator. The gateway receives data packets from all sensor nodes, timestamps and aggregates the incoming measurements, and forwards them to the backend system through an IP-based network. Message Queuing Telemetry Transport (MQTT) is used as the application-layer protocol to ensure lightweight, reliable, and scalable data delivery.

The backend layer provides persistent data storage and real-time visualization capabilities for SHM applications. Incoming sensor data is stored in a database and made available to a web-based dashboard, where vibration signals can be monitored, queried, and analyzed in near real time. This architecture allows seamless integration with higher-level applications, such as structural assessment tools or Digital Twin platforms.

Overall, the proposed SHM-IoT system establishes a complete data pipeline from distributed IMU sensors to web-based monitoring services, while explicitly addressing the requirements of synchronized multi-node operation, TDMA-scheduled wireless communication, and real-time data availability. The detailed design of the sensor nodes, gateway, and backend components is presented in the following subsections.

### 3.2. Sensor Nodes and Data Acquisition

**Figure 2:**
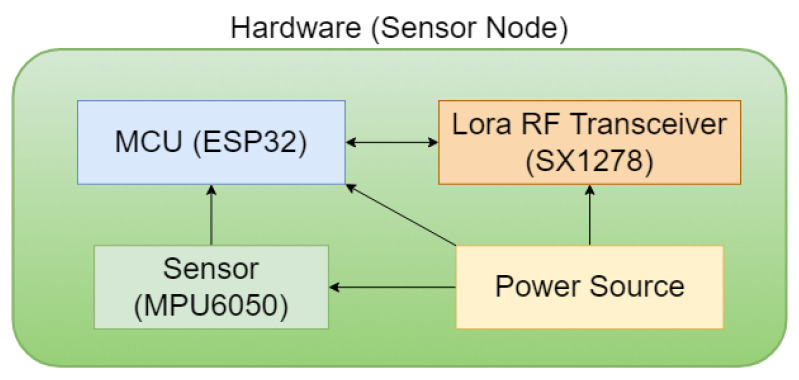
Hardware block diagram of the LoRa IMU sensor node.

Each sensor node in the proposed SHM-IoT system is designed as a low-power embedded device responsible for local vibration data acquisition and periodic wireless transmission. Nodes are deployed at selected locations on the bridge structure to capture dynamic responses under operational and environmental excitations. Figure 2 shows the hardware block diagram of the LoRa IMU sensor node.

From a hardware perspective, the sensor nodes are built around a low-power microcontroller integrated with a LoRa transceiver. An inertial measurement unit (IMU) is connected to the microcontroller to measure multi-axis acceleration of the bridge structure. The MCU coordinates sensor data acquisition, local buffering, and wireless communication through the LoRa module.

The hardware architecture is designed for energy-efficient operation. In particular, the radio module remains in low-power states outside the assigned transmission window, enabling long-term deployment where sensor nodes are expected to operate continuously with limited power resources.

**Figure 3:**
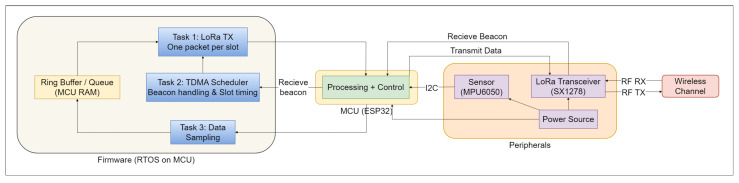
Sensor node hardware–software architecture. IMU data is sampled periodically (5 ms), buffered locally (37-byte payload per sample), and transmitted exactly once in the assigned TDMA slot; non-slot periods keep the radio in receive or sleep to save energy.

Figure 3 summarizes the sensor-node workflow in a concise form. Each node samples the MPU6050 IMU (InvenSense Inc., San Jose, CA, USA) every 5 ms (200 Hz), stores one 37-byte IMU payload in a local buffer, and transmits the buffered payload only during its assigned TDMA slot. This design keeps data acquisition independent from wireless transmission and supports stable multi-node operation.

### 3.3. Master Gateway Architecture

The gateway serves as the central coordination and data aggregation point in the proposed SHM-IoT system. An ESP32-based platform is used as the gateway, interfacing with the LoRa transceiver to receive scheduled uplink data from multiple sensor nodes operating under the TDMA communication scheme. In addition to collecting sensor data, the gateway is responsible for broadcasting periodic synchronization beacons that define the start of each TDMA frame, enabling globally aligned time slots across all nodes.

Upon receiving an uplink packet, the gateway performs lightweight packet validation and attaches metadata—including the reception timestamp and node identifier—without decrypting the sensor payload. This design preserves the end-to-end confidentiality of vibration measurements, as the gateway handles only ciphertext originating from authenticated sensor nodes. The processed packets are subsequently forwarded to the backend infrastructure using the MQTT protocol. MQTT is chosen for its lightweight publish–subscribe model, scalability, and low communication overhead, making it well suited for real-time data streaming in IoT deployments.

The backend infrastructure consists of an MQTT broker, a database server, and a web-based dashboard. Sensor data published by the gateway are subscribed to by backend services, where they are parsed and stored in a database for persistent logging and historical analysis. The database schema is designed to associate sensor measurements with node identifiers and timestamps, enabling efficient data retrieval and long-term monitoring.

On the backend side, an MQTT broker receives the forwarded ciphertext packets, which are then consumed by data ingestion services. These services optionally decrypt the payload, parse the IMU fields, and store the resulting measurements in a dedicated database. Each IMU sample is stored as an individual time-stamped record, enabling efficient retrieval, long-term archiving, and downstream structural analysis. A web-based dashboard provides real-time visualization and historical exploration of vibration data, offering an intuitive interface for monitoring structural conditions.

**Figure 4:**
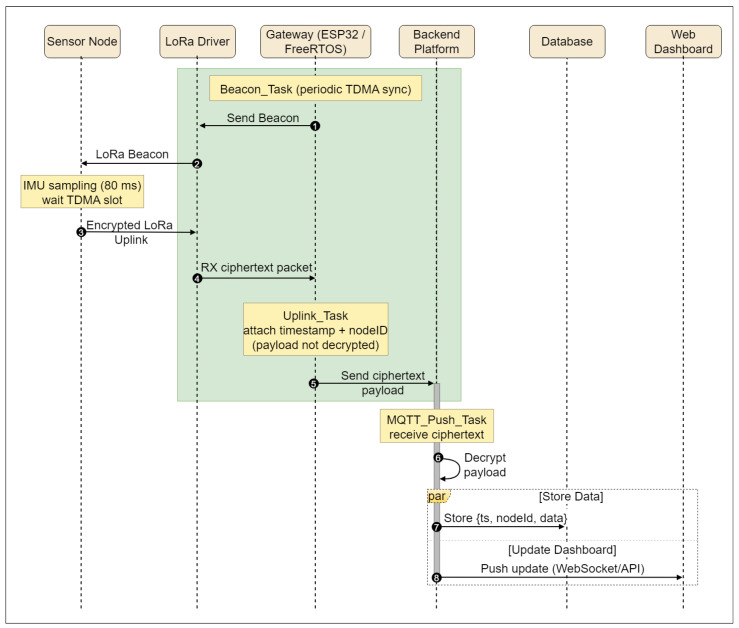
Sequence diagram of gateway system workflow for TDMA-synchronized LoRa uplink, MQTT forwarding, and backend processing.

To illustrate the operational interactions between the sensor nodes, gateway tasks, and backend services, Figure 4 presents the complete end-to-end workflow, from TDMA-synchronized LoRa communication, to FreeRTOS-managed processing within the gateway, to backend storage and dashboard updates. This workflow diagram complements the high-level architecture shown in Figure 1 by detailing the internal task execution and data flow that enable deterministic, real-time monitoring in the proposed SHM–IoT system.

### 3.4. Backend Monitoring Platform

**Figure 5:**
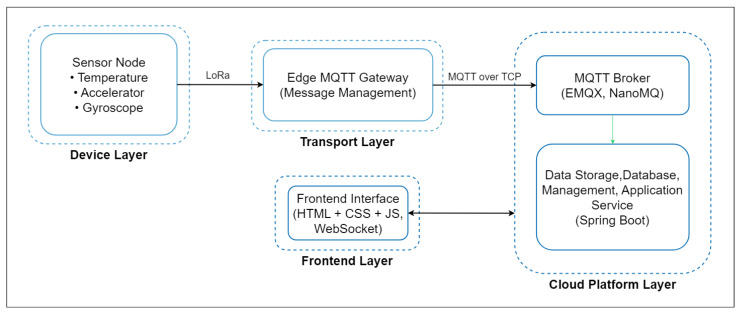
System architecture illustrating four layers: Sensor nodes transmit environmental data via LoRa to an edge MQTT gateway, which pre-processes and forwards it to the cloud via MQTT over TCP. The cloud platform handles data management and visualization, while users interact through a WebSocket-enabled frontend interface.

The backend monitoring platform serves as the central processing and visualization hub within the proposed system. It receives, decrypts, stores, and presents SHM data, thereby enabling comprehensive analysis and informed decision-making. This section details the architectural components, data flow, security mechanisms, data management strategies, and visualization capabilities of the platform. Figure 5 summarizes the four-layer backend monitoring architecture.

#### 3.4.1. System Architecture and Data Ingestion

The backend system is built on a Node.js (v20.11.1) runtime environment with an Express (v4.18.2)-based service layer, providing a scalable and event-driven foundation for data handling. The architecture integrates three primary components: an MQTT-based messaging layer, a database subsystem, and a web-based dashboard.

Data acquisition begins at the gateway, which aggregates TDMA-scheduled uplink transmissions from distributed sensor nodes. Upon reception, each packet is timestamped and annotated with node-specific metadata. Notably, the payload remains encrypted throughout transmission, preserving end-to-end data confidentiality.

The gateway forwards the encrypted data to the backend via the MQTT protocol using a publish–subscribe model. The backend subscribes to relevant topics and asynchronously ingests incoming messages. This lightweight communication mechanism minimizes overhead and is well-suited for real-time IoT deployments with constrained resources.

Upon arrival, each message is passed through a dedicated processing pipeline consisting of decryption, integrity verification, and data parsing stages before being forwarded to storage and visualization modules.

#### 3.4.2. Data Storage and Management

Validated sensor data is persistently stored in a MongoDB database, enabling efficient handling of time-series measurements. Each record includes a server-side timestamp, node identifier, and multi-dimensional IMU measurements such as acceleration and angular velocity. The database uses the following fields, as listed in Table 2.

To support real-time queries and historical analysis, indexing strategies are applied to key fields, including node identifiers and timestamps. This design allows efficient retrieval of recent samples as well as long-term data exploration, which is essential for structural behavior analysis and anomaly detection.

#### 3.4.3. Real-Time Visualization

The platform provides a web-based dashboard implemented using React for real-time monitoring and data exploration. The interface presents both instantaneous sensor readings and recent historical trends through dynamic charts and summary tables.

For each sensor node, multi-axis time-series data is visualized using line charts that simultaneously display acceleration and angular velocity components. Dual-axis representation is used to accommodate differences in scale and units, improving the interpretation of vibration patterns.

**Figure 6:**
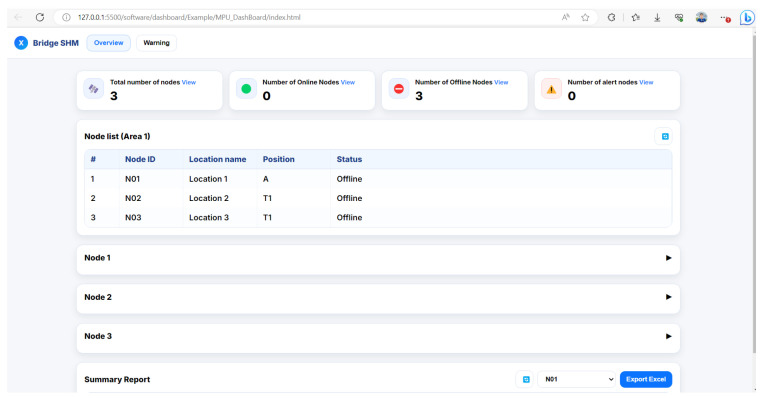
The dashboard was implemented for three nodes.

To ensure responsiveness, the visualization module maintains a bounded data window and incrementally updates using incoming data streams. In addition, a status overview of sensor nodes is provided, enabling quick identification of active or inactive devices within the monitored area. Figure 6 shows the implemented dashboard for the three-node deployment.

### 3.5. Communication Protocol Design

#### 3.5.1. TDMA Frame Structure and Slot Algorithm

To achieve deterministic and collision-free communication among multiple sensor nodes, the proposed system adopts a TDMA-based communication scheme implemented over LoRa. Time is divided into repetitive TDMA frames, each consisting of a synchronization phase followed by a sequence of transmission slots assigned to individual sensor nodes.

**Figure 7:**
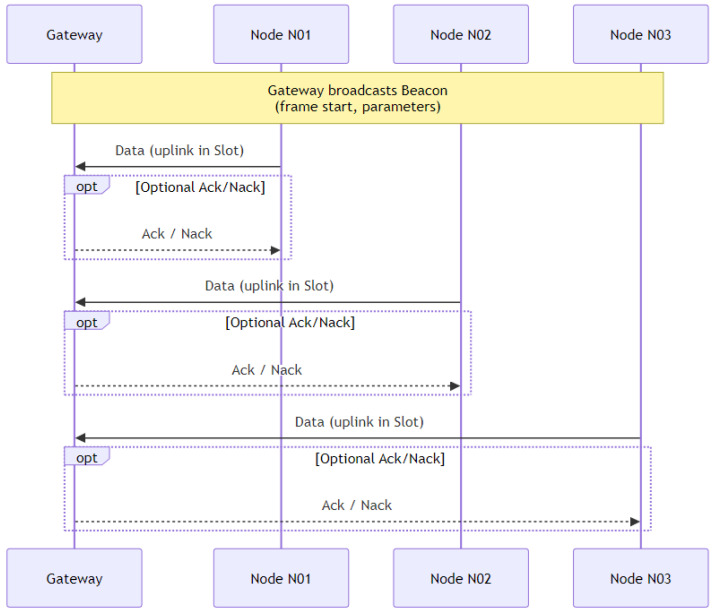
TDMA protocol with gateway-broadcast beacons for software time synchronization; the superframe is partitioned into fixed slots with guard intervals dimensioned from LoRa Time-on-Air and implementation overheads, ensuring non-overlapping transmissions and bounded latency.

At the beginning of each TDMA frame, the gateway broadcasts a LoRa beacon packet that serves as a global time reference for all sensor nodes. Upon reception of the beacon, each sensor node synchronizes its local time and determines the start of the current TDMA frame. Beacon-based synchronization has been widely used to coordinate slot-based transmissions in wireless sensor networks and LoRa-based monitoring systems. Figure 7 illustrates the TDMA protocol with gateway-broadcast synchronization beacons.

Following the beacon, the TDMA frame is partitioned into multiple fixed-length time slots. Each sensor node is statically assigned a unique slot index within the frame. During its assigned slot, a sensor node transmits exactly one data packet containing a single IMU sample with a payload size of 37 bytes. Outside its designated slot, the node keeps the LoRa transceiver in receive or sleep mode to avoid interference with other transmissions and to minimize energy consumption. The slot length is configured to be longer than the worst-case packet transmission time, including the LoRa Time-on-Air and additional guard intervals to compensate for clock drift, synchronization error, and software execution jitter. This ensures that packet transmissions from different nodes do not overlap, even under non-ideal timing conditions. The detailed analysis of LoRa Time-on-Air and its impact on slot configuration is presented in Section 3.5.2.

The TDMA frame length is determined by the number of sensor nodes and the configured slot length. As the number of nodes increases, the frame duration scales approximately linearly because each node requires a dedicated transmission slot. Consequently, the data update interval and end-to-end latency also increase with node count. The current single-channel schedule therefore provides deterministic access within its configured frame capacity, but it should not be interpreted as an experimentally validated solution for arbitrarily large deployments.

The slot scheduling algorithm executed on each sensor node follows a simple and deterministic procedure. After receiving the synchronization beacon, the node computes the start time of its assigned slot based on its slot index and the predefined slot duration. The node then waits until the computed slot boundary and initiates packet transmission. If the beacon is missed, the node refrains from transmitting and waits for the next beacon to re-establish synchronization, thereby preventing unscheduled transmissions.

**Figure 8:**
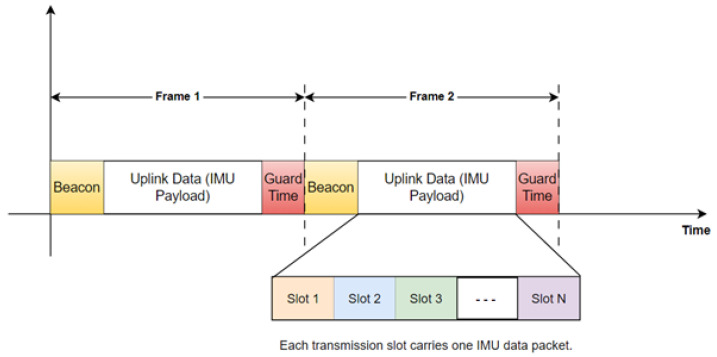
TDMA frame structure with beacon synchronization and uplink transmission slots.

By combining beacon-based synchronization with static slot assignment, the proposed TDMA frame structure provides bounded transmission latency and predictable communication cycles for the tested multi-node SHM deployment. Adaptation to larger bridge deployments would require network-level scaling mechanisms, such as distributing nodes across multiple LoRa channels, dividing the bridge into spatial gateway clusters, or using hierarchical TDMA scheduling with local coordinators. This communication design forms the basis for the real-time software architecture and task scheduling mechanisms described in the subsequent sections. Figure 8 shows the corresponding TDMA frame structure.

#### 3.5.2. LoRa PHY Configuration and Time-on-Air Analysis

Long-range wireless communication between sensor nodes and the gateway is achieved using LoRa technology, which provides low-power and long-distance communication capabilities suitable for IoT monitoring systems [6,7,8]. In a TDMA-based multi-node LoRa network, the achievable number of sensor nodes and the duration of each transmission slot are fundamentally constrained by the packet Time-on-Air (ToA) at the physical layer. Therefore, an analytical evaluation of the LoRa PHY timing characteristics is essential to support bounded scheduling and collision-reduced communication.

LoRa modulation is configured by the Spreading Factor (SF), bandwidth (BW), coding rate (CR), and payload length. These parameters directly affect the symbol duration and the overall packet airtime. The symbol duration $T_{s}$ is given by:

(1) $$T_{s}=\frac{2^{SF}}{BW}$$

An increase in the spreading factor improves link robustness but results in longer symbol duration and higher transmission latency.

Each LoRa packet begins with a preamble used for synchronization and receiver detection. The preamble duration $T_{pre}$ is expressed as:

(2) $$T_{pre}=\left(N_{pre}+4.25\right)\cdot T_{s}$$

where $N_{pre}$ denotes the configured number of preamble symbols, and the constant 4.25 accounts for the synchronization overhead defined in the LoRa PHY specification.

The number of payload symbols $N_{pl}$ depends on the payload size and PHY configuration and is calculated as:

(3) $$N_{pl}=8+\max\left(\left[\frac{8PL-4SF+28+16CRC-20IH}{4(SF-2DE)}\right]\cdot \left(CR+4\right),\;0\right)$$

where PL is the payload length in bytes, CRC indicates whether cyclic redundancy check is enabled, IH denotes the header mode, and DE represents the low data rate optimization flag.

The payload transmission duration $T_{pl}$ is then given by:

(4) $$T_{pl}=N_{pl}\cdot T_{s}$$

The total packet Time-on-Air $T_{ToA}$ is obtained by summing the preamble and payload durations:

(5) $$T_{ToA}=T_{pre}+T_{pl}$$

This value represents the minimum airtime required for a single uplink transmission and serves as the lower bound for TDMA slot allocation.

In practical implementations, additional timing overheads must be considered, including RF transceiver mode switching delays and synchronization uncertainties. Therefore, the TDMA slot length $T_{slot}$ is defined as:

(6) $$T_{slot}=T_{ToA}+T_{RF}+T_{guard}$$

where $T_{RF}$ accounts for transceiver state transitions and $T_{guard}$ is a guard interval used to tolerate clock drift and beacon synchronization errors.

Given a TDMA frame duration $T_{frame}$, the analytically estimated maximum number of schedulable sensor-node slots $N_{max}$ can be expressed as:

(7) $$N_{max}=\left[\frac{T_{frame}}{T_{slot}}\right]$$

This analytical relationship links the LoRa PHY configuration to the number of node slots that can be scheduled within a fixed TDMA frame. The corresponding capacity values are calculated estimates under the assumed TDMA frame length, slot length, guard interval, and LoRa PHY parameters; they should be interpreted as a capacity model for scalability potential rather than as an experimental demonstration of large-scale deployment. By deriving slot durations from PHY-level timing constraints, the proposed system provides deterministic slot planning and bounded frame-level latency for periodic vibration data transmission in structural health monitoring applications.

Based on the analytical TDMA capacity model and the selected LoRa communication parameters, the maximum schedulable network size is estimated from Equation (7) under ideal operating conditions. This value represents a theoretical scheduling capacity rather than an experimentally validated deployment size. The present study experimentally validates only a three-node deployment. Therefore, practical performance for larger networks remains subject to future field validation under realistic wireless channel conditions. It should be emphasized that any estimated node capacity derived from Equation (7) is obtained solely from analytical TDMA frame calculations and assumes stable channel conditions, negligible packet retransmissions, and consistent synchronization performance. Actual network capacity may be lower in practical deployments due to wireless interference, environmental effects, packet loss, and synchronization uncertainties.

Table 3 summarizes the analytically derived packet Time-on-Air and the resulting TDMA slot length for different LoRa PHY configurations. The results show that the maximum schedulable node count depends strongly on the selected LoRa PHY parameters and the fixed TDMA frame duration. This analysis supports the scalability design rationale, whereas physical validation beyond the three-node deployment remains future work.

### 3.6. Security Mechanism

#### 3.6.1. Security Overview

Structural health monitoring (SHM) systems deployed on critical infrastructure, such as bridges, must ensure that collected data is reliable, authentic, and protected from unauthorized manipulation. Since the proposed monitoring platform relies on a distributed wireless sensor network using LoRa communication, the system must address several potential security risks including eavesdropping, message tampering, spoofed devices, and replay attacks.

In the proposed architecture, sensor nodes communicate with an ESP32-based gateway using a TDMA-based LoRa network. Wireless communication channels are inherently vulnerable to interception because transmitted packets can be captured by nearby devices. Furthermore, without proper authentication mechanisms, malicious nodes may attempt to inject fabricated measurements into the network, which could lead to incorrect structural health assessments.

To mitigate these risks, the proposed system integrates a lightweight security mechanism designed for resource-constrained embedded devices. The security architecture focuses on three key properties:

- Confidentiality: Ensuring that transmitted sensor data cannot be interpreted by unauthorized parties.
- Integrity: Guaranteeing that received data have not been modified during transmission.
- Authentication: Verifying that transmitted packets originate from legitimate sensor nodes.

The proposed system implements these protections using symmetric cryptographic primitives optimized for embedded environments. The synchronization beacon is protected using AES-128 ECB for a single fixed-length block, while sensor payloads are protected using AES-CBC with AES-CMAC authentication. The cryptographic operations are implemented using the mbedTLS (v2.28.4) library, which provides hardware-accelerated AES support on the ESP32 platform.

In addition to cryptographic protection, the TDMA communication protocol provides a structural defense against replay and collision attacks. Since each sensor node is assigned a specific transmission slot synchronized through encrypted beacon messages, unauthorized transmissions outside the designated slots can be detected and discarded.

The overall security architecture therefore combines lightweight cryptographic protection with TDMA-based communication control, providing a secure and efficient solution suitable for low-power LoRa sensor networks.

#### 3.6.2. Secure Data Structures

To support secure communication within the TDMA LoRa network, the proposed system defines several protected data structures used for synchronization and sensor data transmission. These structures are designed to maintain compatibility with LoRa packet size limitations while ensuring authentication and confidentiality.

##### Secure TDMA Beacon Structure

Network synchronization is performed through periodic beacon messages transmitted by the gateway. Each beacon contains TDMA scheduling information required for sensor nodes to determine their transmission slots. To protect this synchronization mechanism from spoofing or manipulation, the beacon frame is cryptographically protected before transmission.

The beacon structure consists of 16 bytes, including plain text scheduling information and an authentication tag, as summarized in Table 4.

The cryptographic processing applied to synchronization beacons is formally described in Algorithm 1.
**Algorithm 1** Secure TDMA Beacon Generation and Verification**Input (Gateway):** TDMA parameters, $K_{ENC}$, $K_{MIC}$**Output:** Encrypted TDMA beacon *C*1:Construct plaintext beacon data *M* (12 bytes)2:Compute message integrity code:3:     $MIC\leftarrow CMAC\left(K_{MIC},M\right)\left[0:4\right]$4:Append authentication tag:5:     B←M‖MIC6:Encrypt beacon block:7:     $C\leftarrow AES_{ECB}\left(K_{ENC},B\right)$8:Broadcast ciphertext *C* via LoRa**Input (Sensor Node):** Received beacon *C*, $K_{ENC}$, $K_{MIC}$1:Decrypt beacon:2:     $B\leftarrow AES_{ECB}^{-1}\left(K_{ENC},C\right)$3:Extract *M* and $MIC_{rx}$4:Recompute integrity code:5:     $MIC_{calc}\leftarrow CMAC\left(K_{MIC},M\right)\left[0:4\right]$6:**if** $MIC_{calc}==MIC_{rx}$ **then**7:    Accept beacon and synchronize TDMA scheduler8:**else**9:    Discard beacon10:**end if**

##### Replay Protection

Replay protection is provided through the TDMA synchronization mechanism. Each sensor node is assigned a predefined transmission slot within a synchronized TDMA frame. Packets received outside the expected frame interval or outside the assigned slot window are rejected by the receiver. Consequently, previously captured packets cannot be successfully replayed in subsequent TDMA cycles because they fail temporal validation and slot verification checks.

The MIC is generated using the AES-CMAC algorithm defined in RFC 4493 with a shared symmetric key (MIC_KEY). Let *M* denote the plaintext beacon data. The message authentication code is computed as

(8) $$MIC=\mathrm{CMAC}\left(K_{MIC},M\right)\left[0:4\right]$$

where $K_{MIC}$ is the authentication key and only the first four bytes of the 16-byte CMAC output are used as the MIC.

After the MIC is appended to the beacon data, the entire 16-byte block is encrypted using AES-128 in Electronic Codebook (ECB) mode with the encryption key $K_{ENC}$:

(9) $$C=AES_{ECB}(K_{ENC},M||MIC)$$

where *C* represents the ciphertext transmitted via LoRa.

Although AES-ECB is generally discouraged for multi-block messages because identical plaintext blocks produce identical ciphertext blocks, its use in the proposed synchronization beacon is restricted to a single fixed-length 16-byte block. Since only one encrypted block is transmitted, no inter-block pattern leakage occurs. Furthermore, the beacon payload is protected with an AES-CMAC-based message integrity code prior to encryption. Future implementations may adopt authenticated encryption schemes such as AES-GCM to further strengthen confidentiality and integrity protection.

##### Secure Sensor Data Packet

Sensor measurements are transmitted by nodes during their assigned TDMA slots. Each measurement frame contains vibration data collected from the MPU6050 IMU sensor. To protect the measurement data from interception, the sensor payload is secured using authenticated encryption based on AES-CMAC and AES-CBC before transmission.

The construction of secure sensor data packets is shown in Algorithm 2.
**Algorithm 2** Secure Sensor Data Packet Construction**Input (Sensor Node):** IMU sample *S*, $K_{ENC}$, $K_{MIC}$**Output:** Encrypted sensor payload $IV\|C_{data}$1:Serialize IMU sample into plaintext payload *P*2:Generate initialization vector IV3:Compute message integrity code:4:     $MIC\leftarrow CMAC\left(K_{MIC},P\right)\left[0:4\right]$5:Append authentication tag:6:     $P^{\prime }\leftarrow P\|MIC$7:Encrypt payload:8:     $C_{data}\leftarrow AES_{CBC}\left(K_{ENC},IV,P^{\prime }\right)$9:Transmit $IV\|C_{data}$ during assigned TDMA slot

In this process, the sensor payload is first authenticated using AES-CMAC, ensuring data integrity and source authenticity:

(10) $$MIC=\mathrm{CMAC}\left(K_{MIC},P\right)\left[0:4\right]$$

The plaintext payload and MIC are then encrypted using AES-128 in Cipher Block Chaining (CBC) mode with encryption key $K_{ENC}$ and initialization vector IV:

(11) $$C_{data}=AES_{CBC}\left(K_{ENC},IV,P\|MIC\right)$$

The data encryption process uses the same AES-128 cryptographic primitive implemented through mbedTLS. The sensor data is organized into fixed-size payload blocks that can be encrypted efficiently before LoRa transmission. Since encryption is performed directly on the sensor node, the gateway does not need to access plain text measurements.

As a result, the gateway forwards encrypted payloads directly to the cloud backend, preserving end-to-end confidentiality across the entire monitoring pipeline.

This secure data structure design minimizes overhead while ensuring that both synchronization messages and sensor data are protected from unauthorized modification or inspection.

#### 3.6.3. Secure Communication Flow

**Figure 9:**
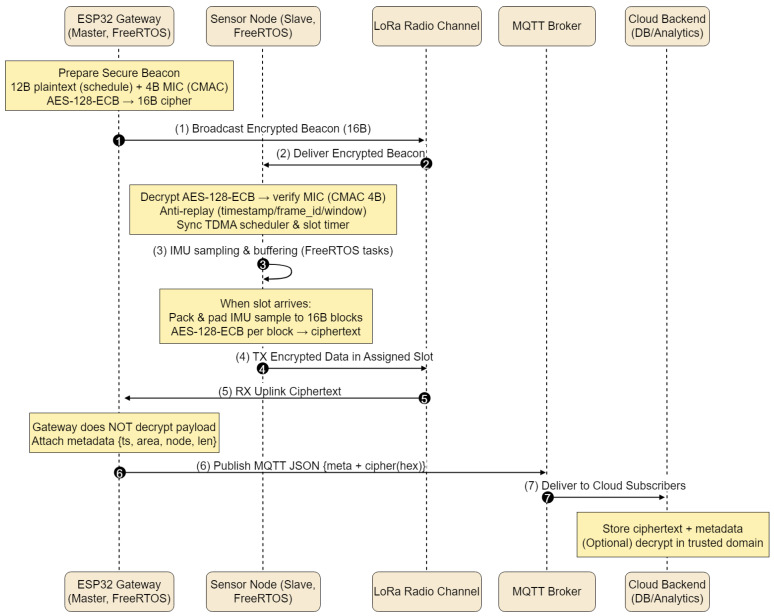
End-to-end secure communication flow: encrypted and authenticated beacon for TDMA sync; encrypted uplink data; ciphertext-only forwarding via MQTT to cloud backend.

The proposed system implements a secure communication workflow that integrates cryptographic protection with TDMA-based wireless communication. The communication process involves three main entities: the gateway, the sensor nodes, and the cloud backend. Security mechanisms are applied at multiple stages to ensure data confidentiality, message integrity, and device authentication. Figure 9 illustrates the end-to-end secure communication flow.

##### Secure Beacon Transmission

The TDMA communication cycle begins with the gateway broadcasting a synchronization beacon that defines the timing of the TDMA frame. The gateway first constructs the plain text beacon structure containing scheduling information required by the sensor nodes.

To protect the beacon from spoofing or manipulation, a message integrity code (MIC) is computed using the AES-CMAC algorithm defined in RFC 4493. Let *M* denote the plain text beacon data and $K_{MIC}$ denote the authentication key. The MIC is calculated as

(12) $$MIC=\mathrm{CMAC}\left(K_{MIC},M\right)\left[0:4\right]$$

where the CMAC output is truncated to four bytes to reduce communication overhead.

After the MIC is appended to the beacon data, the resulting 16-byte block is encrypted using AES-128 in Electronic Codebook (ECB) mode with the beacon encryption key $K_{ENC}$:

(13) $$C=AES_{ECB}(K_{ENC},M||MIC)$$

The encrypted beacon *C* is then transmitted via LoRa to all sensor nodes within the network.

In this implementation, AES-ECB is used only for the fixed-length synchronization beacon, which contains a single 16-byte block after the MIC is appended. Therefore, the well-known ECB weakness associated with repeated patterns across multiple encrypted blocks does not arise for the beacon message. Nevertheless, authenticated encryption schemes such as AES-GCM are considered a stronger option for future implementations.

##### Secure Beacon Reception and Verification

Upon receiving a beacon frame, each sensor node performs a sequence of verification operations before accepting the synchronization information. First, the node decrypts the received ciphertext using the shared encryption key $K_{ENC}$. The plain text beacon data and the MIC field are then extracted.

The node recomputes the CMAC value using the same authentication key $K_{MIC}$ and compares the computed MIC with the received MIC. If the two values match, the beacon is considered authentic and the node synchronizes its TDMA scheduler with the received timing information. Otherwise, the beacon is discarded to prevent malicious synchronization attempts.

##### Secure Sensor Data Transmission

Once synchronized with the TDMA frame, each sensor node periodically collects vibration measurements from the IMU sensor according to the configured sampling interval. The sampled data is temporarily stored in a ring buffer managed by the FreeRTOS-based firmware architecture.

When the node’s assigned TDMA slot begins, the buffered sensor data is encrypted using AES-128 before transmission. The encryption routine is implemented using the mbedTLS cryptographic library, which provides hardware-accelerated AES operations on the ESP32 platform.

The encrypted payload is then transmitted via LoRa during the designated TDMA time slot. Since TDMA schedules only one authorized node within a given slot, packet collisions are reduced and predictable communication timing is supported under the evaluated operating conditions.

##### Secure Data Forwarding to Cloud Backend

The ESP32 gateway receives encrypted packets from sensor nodes during the TDMA communication cycle. Importantly, the gateway does not decrypt the sensor payload. Instead, the encrypted data is forwarded directly to the backend infrastructure, preserving end-to-end confidentiality.

Upon receiving a packet, the gateway encapsulates the ciphertext into a JSON message containing metadata such as timestamp, node identifier, and payload length. The encrypted payload is encoded in hexadecimal format and transmitted to the cloud backend through the MQTT protocol.

This design ensures that sensor measurements remain encrypted throughout the entire data pipeline from the sensor node to the cloud database. As a result, even if intermediate components such as the gateway or network infrastructure are compromised, the confidentiality of the sensor data remains protected.

The proposed security architecture is intended to provide lightweight confidentiality, integrity, and authentication suitable for resource-constrained SHM sensor nodes. The design prioritizes computational efficiency and low communication overhead while acknowledging that advanced security capabilities, including authenticated encryption and full key lifecycle management, remain subjects for future work.

### 3.7. Real-Time Embedded Software Architecture

This section details the real-time firmware architecture that underpins the proposed TDMA-based LoRa SHM system. We articulate the motivation for employing an RTOS, the task model and inter-task pipeline, and the concrete mechanisms that enforce deterministic TDMA slot execution on resource-constrained nodes and the ESP32 gateway. The design aligns with the end-to-end architecture (sensor nodes–LoRa TDMA–ESP32 gateway–MQTT/backend) and the beacon-synchronized MAC described.

#### 3.7.1. Motivation for Using RTOS

Structural health monitoring (SHM) systems based on wireless sensor networks must handle multiple tasks simultaneously, including sensor sampling, data processing, encryption, and wireless communication. In conventional embedded implementations without a real-time operating system (RTOS), these tasks are typically executed sequentially within a single main loop. Such a blocking execution model can introduce several limitations, including unpredictable timing behavior, increased processing latency, and difficulties in maintaining precise communication schedules.

In the proposed TDMA-based LoRa SHM system, deterministic timing is particularly important because each sensor node must transmit data within an assigned time slot. Any delay in sensor processing or communication can lead to slot violations, packet collisions, or frame misalignment. Furthermore, the system must support concurrent operations such as sensor acquisition, cryptographic processing, packet construction, and wireless transmission.

To address these challenges, the embedded firmware adopts the FreeRTOS kernel to provide deterministic task scheduling and concurrent execution of system functions. By decomposing the firmware into multiple tasks with defined priorities, the RTOS enables parallel processing of sensing, encryption, and communication operations. This architecture reduces blocking delays, improves system responsiveness, and ensures that time-critical communication tasks meet their deadlines.

More specifically, it will satisfy the following four real-time goals:

1. Concurrent sensing and communication, allowing sensor acquisition, data processing, and wireless transmission to operate in parallel without blocking the processing pipeline.

2. Deterministic TDMA slot execution, ensuring that each node transmits data within its assigned time slot with bounded timing deviation.

3. Reduced end-to-end processing latency, achieved by overlapping sensing, encryption, and communication stages through a task-based pipeline.

4. Robust responsiveness to asynchronous events, such as radio interruptions, synchronization beacons, and backend communication triggers.

FreeRTOS provides the necessary mechanisms—including fixed-priority preemptive scheduling, software timers, and queue-based inter-task communication—to support these real-time requirements. The resulting task structure and scheduling strategy used to achieve these goals are described in the following subsection.

#### 3.7.2. RTOS Task Model

The embedded firmware is structured as a set of independent tasks scheduled by the FreeRTOS kernel. Each task is responsible for a specific functional component of the system, such as sensor acquisition, communication scheduling, data encryption, or network transmission. Tasks are assigned priorities according to their real-time requirements, ensuring that time-critical operations are executed without delay.

##### Node-Side Tasks

Each sensor node executes five core tasks, forming the real-time pipeline for sensing, securing, and transmitting vibration data:

- IMU Sampling Task (periodic). Samples 3-axis acceleration every 5 ms (200 Hz), storing results in a bounded ring buffer. Because sampling is decoupled from communication timing, the sampling rate remains stable even if slot boundaries shift slightly between TDMA frames.
- TDMA Scheduler Task (periodic per frame). Processes gateway synchronization beacons, aligns the local clock to the TDMA frame epoch, and computes the transmission slot start time. It then configures a high-resolution FreeRTOS software timer to release the transmission task precisely at the slot boundary.
- LoRa TX Task (event-driven, high priority). Activated only within the assigned slot window. It retrieves one IMU sample (37-byte payload before security encapsulation), transmits it over LoRa, and returns the radio to RX or sleep mode. Outside the slot, the task remains dormant to prevent unscheduled transmissions.

**Figure 10:**
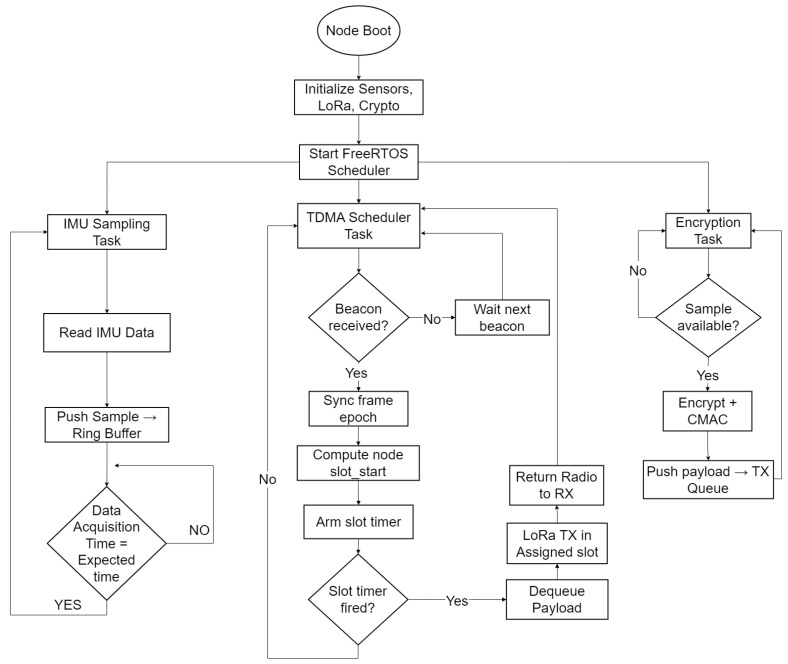
Flowchart of slave node real-time task execution under FreeRTOS.

Figure 10 summarizes the slave-node real-time task execution flow.

Encryption/Integrity Task (deferred). Performs AES-128 encryption and CMAC-based integrity protection for IMU samples before transmission. Executed as a medium-priority deferred task, it prevents cryptographic overhead from introducing jitter into the real-time radio path.

##### Gateway-Side Tasks

The ESP32-based master gateway runs a complementary set of FreeRTOS tasks that support system-wide TDMA synchronization and backend forwarding:

- Beacon Generation Task (periodic). Emits synchronization beacons at the beginning of each TDMA frame. As beacon timing defines the global time reference for all nodes, this task runs with high priority to minimize jitter.
- LoRa RX Task (high priority). Handles packet reception during node transmission slots. It appends metadata (timestamp, node ID) and forwards packets into a queue for backend processing. Payloads remain encrypted end-to-end, minimizing gateway processing overhead.
- MQTT Publisher Task (low/medium priority). Forwards received ciphertext packets to the backend server using MQTT. Since this task has the lowest timing criticality, it is intentionally scheduled at low priority to avoid any impact on beacon timing or LoRa reception windows.

**Figure 11:**
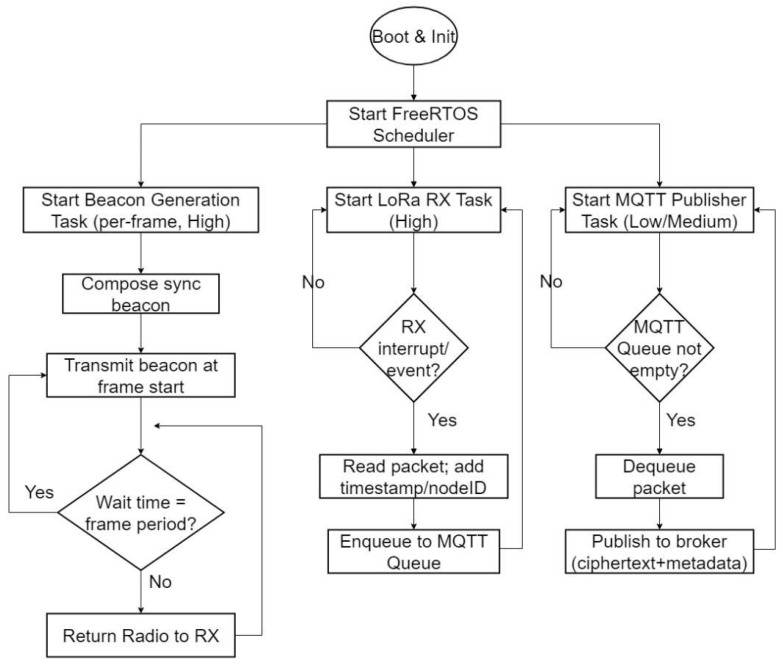
Flowchart of gateway real-time task execution under FreeRTOS.

Figure 11 summarizes the gateway real-time task execution flow.

The major tasks were called above and implemented in the system are summarized in Table 5.

#### 3.7.3. Communication Pipeline

To prevent timing interference between sensing, communication, and backend data handling, the system adopts a pipelined communication architecture with explicit data flow isolation. The transmission task accesses this buffer exclusively during the assigned TDMA slot, avoiding contention with the sampling task.

On the gateway, LoRa reception tasks operate independently of IP-based network communication. Received packets are placed into intermediate queues and subsequently processed by MQTT publishing tasks running at lower priority. This design prevents delays in backend communication from affecting real-time LoRa reception or beacon generation.

The use of task queues and inter-task synchronization mechanisms provided by FreeRTOS enables clear separation between real-time radio operations and non-real-time backend communication. As a result, variability in MQTT transmission latency or web-based data visualization does not propagate back into the time-critical LoRa communication pipeline.

#### 3.7.4. Deterministic Slot Execution on Sensor Nodes

Deterministic execution of TDMA slots on sensor nodes is achieved by combining beacon-based time synchronization, software timers, and priority-controlled task activation. A LoRa synchronization beacon at the beginning of each TDMA frame, establishing a global time reference shared by all sensor nodes.

Upon receiving the beacon, each node aligns its local frame counter and computes the start time of its assigned transmission slot according to

(14) $$t_{slot}=t_{epoch}+i\cdot T_{slot}$$

where $t_{epoch}$ denotes the beacon reception time, i∈N denotes the node’s assigned slot index within the TDMA frame and $T_{slot}$ represents the configured slot duration. A high-resolution FreeRTOS software timer is then programmed to trigger the LoRa TX task precisely at this computed slot boundary. If a beacon is missed, the node refrains from transmitting until synchronization is restored by a subsequent beacon, thereby preventing unscheduled transmissions and potential collisions.

To further guarantee reliable slot execution, the slot duration is selected based on the worst-case LoRa packet Time-on-Air (ToA), including radio state transition delays and an additional guard interval. This guard time compensates for oscillator drift, synchronization errors, and potential software scheduling jitter. The detailed ToA analysis used to determine slot duration is presented in Section 3.5.2 and summarized in Table 3.

The timing accuracy and deterministic slot execution achieved by this design are quantitatively evaluated in Section 4.

## 4. Experimental Evaluation

This section presents the experimental validation of the proposed TDMA-based LoRa SHM–IoT system. The experiments aim to demonstrate the correct operation of the end-to-end architecture described in Section 3 and the FreeRTOS-based real-time software design presented in Section 4. In particular, the results validate the integration of sensing, TDMA-scheduled wireless communication, backend data storage, and real-time visualization.

### 4.1. Experimental Setup

#### 4.1.1. Hardware Platform

The experimental system consists of multiple IMU-based sensor nodes (three nodes in the current experimental setup) and a central LoRa gateway. Both the sensor nodes and the gateway are implemented using the ESP32-WROOM-32D module, which integrates a dual-core ESP32 microcontroller. This platform provides sufficient computational capability and communication flexibility for IoT-based structural health monitoring (SHM) applications while supporting real-time task execution under FreeRTOS.

Although only three physical sensor nodes were deployed, the selected configuration exercises all key system functions required for multi-node operation, including beacon-based synchronization, slot allocation, collision-free communication, gateway aggregation, backend data handling, and dashboard visualization. The purpose of the experiment is therefore architectural validation rather than empirical determination of the maximum network capacity.

##### Gateway Hardware and Core Assignment

On the gateway side, the dual-core architecture of the ESP32 is explicitly exploited to separate time-critical LoRa communication tasks from network-related processing. In the proposed implementation, Core 0 is primarily responsible for networking functions, including WiFi connectivity, TCP/IP stack management, and MQTT data transmission to the remote monitoring server. Core 1 executes real-time communication functions, including LoRa driver operations, reception of TDMA-scheduled uplink packets from sensor nodes, and synchronization beacon transmission. This separation minimizes timing interference between IP-based networking and LoRa communication, improving timing predictability and reducing jitter in TDMA frame execution.

##### Sensor Node Hardware and Task Execution

On the sensor nodes, the ESP32-WROOM-32D module is used with a simplified task configuration compared to the gateway. Sensor nodes do not execute WiFi or TCP/IP tasks. Instead, both cores are dedicated to real-time functions, including IMU data acquisition, TDMA slot scheduling, and LoRa transmission. This configuration ensures that sensing and communication tasks are executed with predictable timing and minimal overhead, which is essential for accurate synchronization under the TDMA scheme.

##### Hardware Specifications

Sensor Nodes

- MCU: ESP32-WROOM-32 (Espressif Systems (Shanghai) Co., Ltd., Shanghai, China);
- IMU: MPU6050 (triaxial accelerometer and gyroscope);
- LoRa Transceiver: SX1278 (Semtech Corporation, Camarillo, CA, USA) (434 MHz);
- Power: 18650 Li-ion battery (manufacturer not specified; sourced locally, Hanoi, Vietnam) via CN3060 charging module.

Gateway

- MCU: ESP32-WROOM-32 (Espressif Systems (Shanghai) Co., Ltd., Shanghai, China);
- LoRa Transceiver: SX1278 (Semtech Corporation, Camarillo, CA, USA) (433.5 MHz);
- Power: 18650 Li-ion battery (manufacturer not specified; sourced locally, Hanoi, Vietnam) via CN3060 charging module.

**Figure 12:**
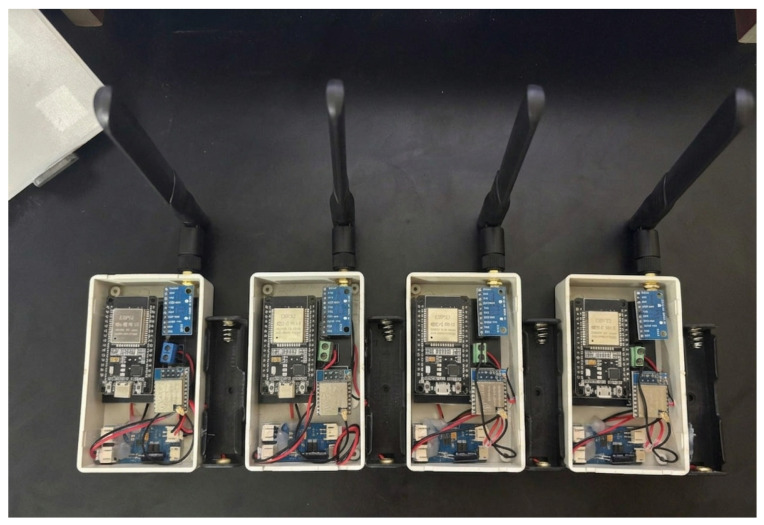
Developed sensor node hardware used in the experimental setup.

Figure 12 shows the developed sensor node hardware used in the experimental setup.

#### 4.1.2. Software and Firmware Architecture

This subsection reports the experimental firmware implementation and radio configuration used for validation, while the operating principles of the task model and TDMA protocol are described in the system-design section.

##### Sensor Nodes

The sensor-node firmware implementation follows the architecture and task model presented earlier. In this experiment, the implementation verifies the practical realization of the proposed sensing, security, and TDMA scheduling pipeline on the ESP32 platform.

The uplink LoRa configuration used in the experiment is a carrier frequency of 434 MHz, spreading factor SF = 9, bandwidth BW = 125 kHz, coding rate CR = 4/5, and payload length PL = 48 bytes. The packet Time-on-Air is computed using Equations (1)–(7), yielding:

$$T_{ToA}\approx 345\mathrm{ms}$$

##### Gateway Nodes

The gateway firmware is implemented according to the gateway architecture described earlier and is evaluated here in terms of its experimental synchronization and forwarding configuration.

For synchronization, the beacon signal uses a carrier frequency of 433.5 MHz, spreading factor SF = 10, bandwidth BW = 250 kHz, coding rate CR = 4/5, payload length PL = 16 bytes, and a preamble length of 12 symbols. Using the same Time-on-Air formulation, the beacon airtime is approximately:

$$T_{ToA}\approx 82\mathrm{ms}$$

##### TDMA Configuration

Based on the measured implementation settings and uplink airtime, the experimental slot duration is configured as:

$$T_{slot}\approx 360\mathrm{ms}$$

This value includes margins for RF transceiver switching delay and synchronization uncertainty. Together, these settings define the implementation parameters used in the experiments and complement the TDMA operating procedure specified in the system architecture.

#### 4.1.3. Deployment Environment

Experiments were conducted indoors across a laboratory space, with sensor nodes positioned 5–15 m apart. The gateway was located centrally to ensure equal reception quality. All measurements reflect real hardware behavior.

### 4.2. RTOS Task Timing Analysis

This experiment evaluates the execution-time behavior of key FreeRTOS tasks on both the gateway and sensor nodes, with the objective of determining whether the system provides bounded and predictable timing suitable for a soft real-time TDMA-based LoRa communication architecture. The analysis examines essential runtime metrics—including average, worst-case, and best-case execution times, as well as jitter and standard deviation—to assess temporal stability and ensure tasks complete within their deadlines.

- Average execution time (Avg);
- Worst-case execution time (WCET);
- Best-case execution time (BCET);
- Jitter, defined as the difference between the maximum and minimum observed execution times;
- Standard deviation (StdDev) to evaluate execution variability.

To obtain precise measurements, each task was instrumented at its entry and exit points using a microsecond-resolution hardware timer integrated into the ESP32 platform. This method ensures accurate capture of fine-grained execution intervals, including interrupt-driven behaviors and variations introduced by concurrent tasks. For each evaluated task, 1000 consecutive samples were recorded under normal operating conditions, providing a statistically meaningful dataset that reflects realistic system dynamics.

Based on the collected dataset, several timing metrics were computed to characterize task-level behavior.

The average execution time is defined as:

(15) $$T_{avg}=\frac{1}{N}\sum_{i=1}^{N}T_{i}$$

The worst-case and best-case execution times are given by:

(16) $$T_{WCET}=\max\left(T_{i}\right),\;T_{BCET}=\min\left(T_{i}\right)$$

The jitter is computed as:

(17) $$J=T_{WCET}-T_{BCET}$$

The standard deviation is defined as:

(18) $$\sigma =\sqrt{\frac{1}{N}\sum_{i=1}^{N}{\left(T_{i}-T_{avg}\right)}^{2}}$$

To evaluate execution stability, the coefficient of variation (CV) is used:

(19) $$CV=\frac{\sigma }{T_{avg}}\times 100\%$$

#### 4.2.1. Gateway-Side Evaluation

On the gateway side, these performance metrics are used to characterize the real-time execution properties of the corresponding tasks, including:

- TDMA Scheduler (Beacon Generation) Task;
- LoRa RX Task;
- MQTT Publisher Task.

In Table 6, the TDMA Scheduler Task shows an average execution time of 4631.212 μs, which remains well below its activation period, ensuring that scheduling operations always complete before the next scheduling cycle. No deadline misses were observed during the acquisition of 1000 consecutive data samples, as shown in Table 6, confirming that all tasks consistently met their timing constraints under the TDMA-based scheduling framework. Specifically, the worst-case execution time (WCET) of 5046 μs is significantly smaller than the task period. Although the jitter reaches 1600 μs, this value represents only a small fraction of the TDMA slot duration (e.g., 80 ms), meaning that such variability does not impact slot alignment or synchronization. Thus, the scheduler maintains reliable frame-level coordination despite moderate timing fluctuations.

The LoRa RX Task exhibits the most deterministic behavior, with an average execution time of 951.96 μs and a WCET of 1019 μs. Its small jitter of 297 μs and minimal standard deviation confirm a highly stable execution profile. This ≈1 ms processing time is effectively near-real-time and crucial for handling radio interrupts. No packet loss due to processing delay was observed during the experiment. The task’s tight execution bounds ensure that incoming packets are processed consistently and well within the timing constraints of the TDMA uplink.

In contrast, the MQTT Publisher Task shows notable variability, with execution times ranging from 2268 μs to 8669 μs. This jitter is expected due to the nondeterministic nature of Wi-Fi and TCP/IP communication. However, because the task is assigned the lowest priority, its timing fluctuations do not propagate into the time-critical TDMA communication pipeline, ensuring that real-time performance remains unaffected.

At the system level, the measurements confirm a clear isolation between time-critical and non-critical tasks. This architectural separation ensures that deterministic TDMA operation is preserved even in the presence of network-layer variability. These results validate that the proposed system achieves bounded execution time and temporal predictability, which are essential requirements for real-time TDMA-based communication systems.

#### 4.2.2. Sensor Node Evaluation

On the sensor nodes, these performance metrics are used to characterize the real-time execution properties of the corresponding tasks, including:

- IMU Sampling task;
- LoRa Processing task;
- Encryption task.

Based on the data in Table 7 and Formula (19), the coefficient of variation is computed as:

$$CV_{IMUSampling}=\frac{StdDev_{IMUSampling}}{Avg_{IMUSampling}}=\frac{31.82922626}{2668.434}\approx 1.19\%$$

The IMU Sampling task exhibits an average execution time of 2668.434 μs, with a WCET of 3623 μs and a BCET of 2649 μs, resulting in a jitter of 974 μs. Despite this relatively large jitter, the standard deviation remains low at 31.82922626 μs, corresponding to a low coefficient of variation of approximately 1.19%. This indicates that execution times are tightly clustered, and the observed variability is primarily due to infrequent outliers rather than continuous fluctuations.

The CV of the encrypt task was also calculated:

$$CV_{Encryption}=\frac{StdDev_{Encryption}}{Avg_{Encryption}}=\frac{10.79077157}{102.307}\approx 10.55\%$$

The Encryption task exhibits a relatively high coefficient of variation (10.55%), mainly due to its short execution time scale. Despite this, the absolute latency remains below 0.5 ms, indicating that its variability has minimal impact on the overall system timing. The observed jitter is therefore acceptable and does not compromise real-time performance.

And on the LORA_TX task:

$$CV_{LoraProcessing}=\frac{StdDev_{LoraProcessing}}{Avg_{LoraProcessing}}=\frac{85.27946829}{2720.267}\approx 3.13\%$$

In contrast, the LoRa Processing task demonstrates low variability (CV = 3.13%) with tightly bounded execution time. This predictable behavior is critical for TDMA scheduling, as it ensures that packet transmission consistently occurs within the assigned time slot. Consequently, the task provides sufficient timing reliability to prevent slot violations and supports stable multi-node communication.

#### 4.2.3. Pipeline-Level Analysis

The total average execution time of the processing pipeline (SENSOR → ENCRYPT → LORA_TX) is:

$$T_{total}=\sum_{k=1}^{3}T_{avg,k}=5491.008\;\mu s$$

The worst-case execution time of the pipeline is:

$$T_{WCET}^{total}=3623+443+3011=7077\;\mu s$$

The best-case execution time is:

$$T_{BCET}^{total}=2649+99+2648=5396\;\mu s$$

Thus, the pipeline jitter is:

(20) $$J_{pipeline}=7077-5396=1681\;\mu s$$

The measured pipeline execution time, even in the worst case ($T_{WCET}^{total}=7.08$ ms), is significantly smaller than the LoRa packet Time-on-Air ($T_{ToA}\approx 345$ ms) derived in Section 3.5.2. This indicates that the system is not computation-bound, but rather constrained by the wireless transmission latency at the physical layer. The timing margin confirms that local sensing, encryption, and packet-preparation tasks do not dominate the TDMA slot duration in the tested three-node deployment. Estimated larger node counts, including values derived from the TDMA capacity model, should therefore be interpreted as analytical scheduling-capacity estimates rather than experimentally validated network sizes.

Furthermore, when compared to the configured TDMA slot duration ($T_{slot}\approx 360$ ms), the processing pipeline occupies less than 2% of the available slot time. As a result, all sensing, encryption, and packet preparation operations are completed well in advance of the assigned transmission slot.

This timing relationship ensures that each sensor node can reliably buffer its data and transmit within the allocated TDMA slot without risk of overrun. Moreover, the large timing margin between processing completion and transmission time provides robustness against execution jitter ($J_{pipeline}=1.68$ ms) and minor synchronization uncertainties.

Overall, these results confirm that the proposed system is communication-dominated, where the LoRa Time-on-Air dictates the system timing, while the processing pipeline introduces negligible delay and does not impact TDMA scheduling determinism.

### 4.3. TDMA Slot Timing Accuracy and Jitter

To evaluate the timing precision of TDMA slot execution, the slot timing error is defined as the difference between the actual transmission time and the expected slot start time:

(21) $$\Delta t_{i}=t_{actual,i}-t_{expected,i}$$

where $t_{expected,i}$ is derived from beacon synchronization and slot scheduling, and $t_{actual,i}$ is recorded at the moment the transmission task is triggered.

Based on the collected samples, the following statistical metrics are computed.

Mean timing error:

(22) $$\Delta t_{avg}=\frac{1}{N}\sum_{i=1}^{N}\Delta t_{i}$$

Maximum and minimum deviation:

(23) $$\Delta t_{max}=\max\left(\Delta t_{i}\right),\;\Delta t_{min}=\min\left(\Delta t_{i}\right)$$

Jitter (peak-to-peak variation):

(24) $$J=\Delta t_{max}-\Delta t_{min}$$

Standard deviation:

(25) $$\sigma =\sqrt{\frac{1}{N}\sum_{i=1}^{N}{\left(\Delta t_{i}-\Delta t_{avg}\right)}^{2}}$$

These metrics collectively characterize the determinism and stability of the execution of the TDMA slot. For timing tables with 1000 samples, 95% confidence intervals are calculated as $\bar{x}\pm 1.96\sigma /\sqrt{N}$ and reported to quantify uncertainty in the mean execution time.

For each node, a total of 1000 timing error samples are continuously monitored and recorded on the Lora transmit task. The statistical results computed from this dataset are summarized in Table 8.

The results in Table 8 indicate high timing determinism, with a mean error of 3.04 μs and a very low standard deviation of 0.6 μs. Although the maximum deviation reaches 11 μs, this peak occurs only during the system startup phase and can be attributed to transient synchronization and scheduling alignment effects. After initialization, the timing error stabilizes around 3 μs, with some of 4 μs leading to a bounded jitter of 8 μs.

This level of timing variation is negligible relative to the TDMA slot duration, ensuring reliable slot alignment and preventing inter-slot interference. Overall, the results confirm that the proposed TDMA mechanism provides stable and predictable timing behavior under steady-state operation.

### 4.4. End-to-End Latency of the Data Pipeline

The objective of this experiment is to quantitatively evaluate the real-time performance of the proposed SHM–IoT system by measuring the end-to-end latency across the complete data pipeline, from IMU data acquisition at the sensor node to final data storage in the backend database.

To ensure statistical reliability, the measurements were conducted over 1000 consecutive data samples under normal operating conditions. Timestamp markers were inserted at key stages of the pipeline, as described in Section 3, and the latency of each stage was computed as the time difference between consecutive timestamps.

The evaluation focuses on the following latency components:

- Sensor processing latency: measured from IMU data acquisition to the completion of data encryption.
- Encryption-to-transmission latency: defined as the interval between the end of encryption and the start of LoRa transmission.
- LoRa transmission latency: corresponding to the packet Time-on-Air (ToA), i.e., the duration from transmission start at the sensor node to successful reception at the gateway.
- Gateway processing latency: calculated from packet reception at the gateway to the initiation of MQTT publishing.
- MQTT transport latency: measured between MQTT publish initiation at the gateway and message reception at the backend server.
- Database latency: measured from the start of data insertion to the completion of the database commit operation.

The total end-to-end latency is computed as the sum of all stage-wise delays:

(26) $$T_{E2E}=\sum_{i=1}^{N-1}\left(t_{i+1}-t_{i}\right)$$

where $t_{i}$ denotes the timestamp recorded at the *i*-th stage of the pipeline.

The aggregated latency results, averaged over 1000 samples, are summarized in Table 9.

The results in Table 9 show that the end-to-end latency is dominated by the LoRa transmission stage, which contributes the majority of the total delay due to its long Time-on-Air.

The sensor node processing latency ranges from 0.89 ms to 10.934 ms, indicating bounded variation caused by RTOS scheduling and buffering. Despite this, the processing delay remains small compared to the overall latency and does not represent a system bottleneck.

The gateway and backend latencies are relatively stable and contribute only a minor portion to the total delay.

Overall, the system exhibits a bounded end-to-end latency of approximately 390–410 ms, confirming predictable performance suitable for TDMA-based low-frequency SHM applications. The results also indicate that the system is communication-bound, with optimization primarily dependent on LoRa PHY parameters.

### 4.5. Packet Delivery Ratio

Packet Delivery Ratio (PDR) is a key performance metric used to evaluate the reliability of the communication system. It represents the ratio of successfully received packets at the gateway to the total number of packets transmitted by sensor nodes. A high PDR indicates robust and reliable data transmission, which is essential for real-time structural health monitoring (SHM) applications.

To evaluate PDR, each sensor node appends a monotonically increasing sequence ID to every transmitted packet. At the gateway, the received sequence IDs are tracked for each node individually. Packet loss is determined by detecting gaps in the sequence numbers.

For a given node, the PDR is calculated as:

(27) $$PDR=\frac{N_{\mathrm{received}}}{N_{\mathrm{received}}+N_{\mathrm{lost}}}\times 100\%$$

where $N_{\mathrm{received}}$ is the number of successfully received packets and $N_{\mathrm{lost}}$ is the number of missing packets inferred from sequence gaps.

The system runs continuously for a fixed duration, and PDR statistics are collected at the gateway for each node.

Table 10 summarizes the PDR performance for multiple sensor nodes.

The experimental results show that Node 1 achieves a PDR of 96.93% with 35 lost packets out of 1141 transmissions, while Node 2 and Node 3 achieve higher PDR values of 98.69% and 98.77%, respectively, with significantly fewer packet losses. The average PDR of the system is approximately 98.13%, indicating high reliability in multi-node wireless communication.

The variation between nodes is relatively small (about 1.84%), demonstrating consistent performance across the network. The slightly lower PDR observed in Node 1 may be attributed to environmental factors, such as signal attenuation or minor timing deviations.

Overall, the high and stable PDR confirms that the integration of RTOS-based scheduling and TDMA communication supports predictable transmission, minimizes packet collisions in the tested setup, and maintains reliable data delivery.

However, it should be noted that the PDR may still be affected by external factors such as transmission power, communication range, and environmental interference. Under suboptimal conditions, signal attenuation may increase packet loss. Nevertheless, within the tested setup, the system demonstrates strong robustness and is well-suited for real-time SHM applications.

### 4.6. Comparison with Non RTOS Baseline

The objective of this evaluation is to quantify the performance improvements achieved by the proposed FreeRTOS-based TDMA architecture compared to a baseline implementation without real-time scheduling. In particular, the comparison focuses on timing determinism, execution stability, and communication reliability under identical operating conditions.

To ensure a fair comparison, two firmware implementations were developed and tested on the same hardware platform. The sensor-node latency comparison is summarized in Table 11:

- Baseline (Non-RTOS Loop and Non Time Distribution): A bare-metal sequential design in which sensor sampling, data processing, and LoRa transmission are executed in a single loop without task separation or scheduling.
- Proposed System (FreeRTOS + TDMA): A multi-task architecture where sensing, processing, and communication are executed as independent tasks under FreeRTOS, combined with a TDMA-based communication scheme to ensure collision-free transmission.

From the comparison table, it can be seen that the execution time of each task between the NON-RTOS and RTOS implementations is nearly identical, since both run on the same hardware architecture. For example, sensor read time is almost the same in both NON-RTOS and RTOS around 2668 µs in NON-RTOS versus 2670 µs in RTOS, with RTOS showing some outliers. However, the RTOS allows tasks to run concurrently instead of waiting sequentially, avoiding the blocking behavior inherent in the single-loop structure of NON-RTOS. As a result, RTOS improves the overall system latency and provides better determinism during communication operations.

Both systems were evaluated using identical sensor configurations, transmission parameters, and sampling intervals. Performance metrics were extracted from system logs, including loop timing, end-to-end latency, jitter, and packet reliability. Table 12 compares the main timing and transmission parameters.

The FreeRTOS + TDMA system demonstrates clear advantages over the NON-RTOS baseline:

- Deterministic Task Scheduling: Independent tasks prevent blocking, reducing the occurrence of latency spikes seen in the superloop approach.
- Stable Communication: TDMA eliminates collisions, leading to higher and more consistent Packet Delivery Ratio (PDR), improving reliability especially under multi-node conditions.
- Predictable Timing: Loop drift and queue-induced delays are effectively eliminated, allowing tasks such as LoRa transmission and sensor sampling to occur at precise intervals.
- Scalability Potential: The RTOS + TDMA architecture provides a predictable scheduled communication framework whose network capacity can be analytically estimated using the TDMA timing model presented in Section 3.5. However, large-scale deployments involving substantially more nodes were not experimentally evaluated in the present study and therefore remain future work.
- End-to-End Performance: Total pipeline latency is smoother, and jitter is significantly reduced, providing higher confidence in real-time data delivery for downstream processing.

In summary, the FreeRTOS + TDMA design outperforms the NON-RTOS baseline in timing determinism, execution stability, and communication reliability, validating the proposed architecture for real-time sensor networks.

### 4.7. Comparison with a Wired Reference Measurement System

To validate the measurement accuracy and practical applicability of the proposed TDMA-based LoRa IoT vibration sensing system, experimental results were compared against those obtained from a commercial wired vibration measurement system. The wired system serves as a laboratory-grade reference instrument and is widely used in structural vibration measurement and dynamic analysis.

The reference system employed in this study is the IMV VM-5112 servo-type vibration meter manufactured by IMV Corporation, Osaka, Japan, interfaced with a monitoring laptop for real-time waveform visualization and data analysis. The system utilizes IMV VP-5112 servo-type acceleration pickup heads, which provide high accuracy and stability in low-frequency vibration measurements, including acceleration, velocity, displacement, and tilt.

**Figure 13:**
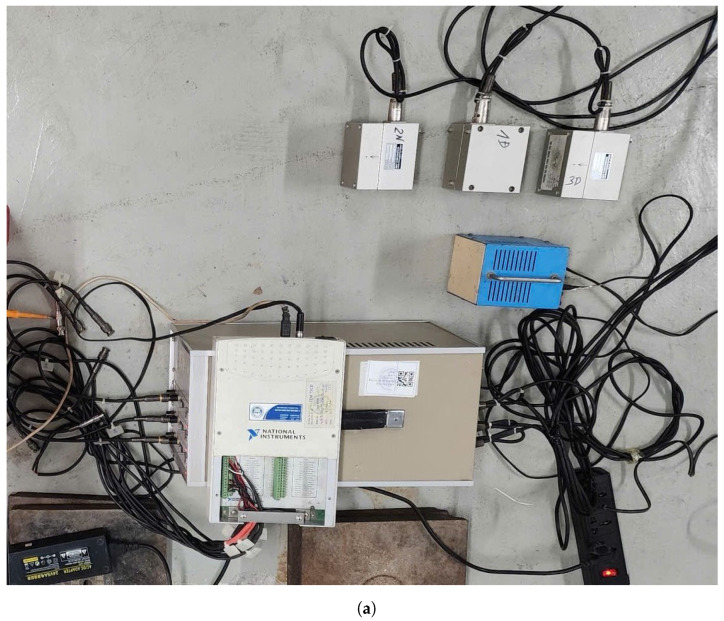

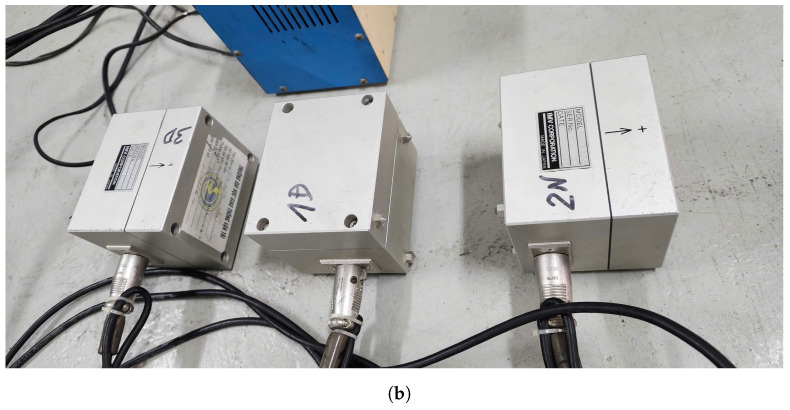
IMV VM-5112 servo-type vibration measurement system used in the experiments. (**a**) Overview of the complete wired reference measurement setup, comprising the VM-5112 vibration meter, signal conditioning units, power supply, and associated cabling. (**b**) Close-up view of the servo-type acceleration pickup heads (IMV VP-5112), illustrating the sensing elements used for vibration measurements.

Figure 13 illustrates the wired reference measurement setup used during the laboratory experiments. Figure 13a presents an overview of the complete measurement system, including the VM-5112 vibration meter, signal conditioning and amplification units, power supply, and cabling. Figure 13b shows a close-up view of the servo-type acceleration pickup heads (VP-5112) employed for vibration data acquisition.

The commercial reference system relies on direct wired connections between the acceleration pickup heads, the vibration meter, and the data monitoring equipment. This configuration enables high-bandwidth signal transmission with negligible communication delay and provides highly accurate measurements. However, it requires fixed cabling, manual installation, and reconfiguration for each measurement campaign, limiting its suitability for long-term or large-scale deployments.

In contrast, the proposed system adopts a wireless IoT-based architecture, where sensor nodes equipped with embedded accelerometers are permanently installed on the structure. Vibration data is transmitted wirelessly to the gateway using a TDMA-scheduled LoRa communication framework under FreeRTOS control. While this approach introduces higher communication latency and lower sampling rates compared to the wired reference system, it enables continuous, real-time, and unattended structural monitoring without the need for extensive cabling or repeated on-site installation.

Table 13 summarizes the key technical differences between the commercial wired reference system and the proposed TDMA-based LoRa IoT sensing system.

**Figure 14:**
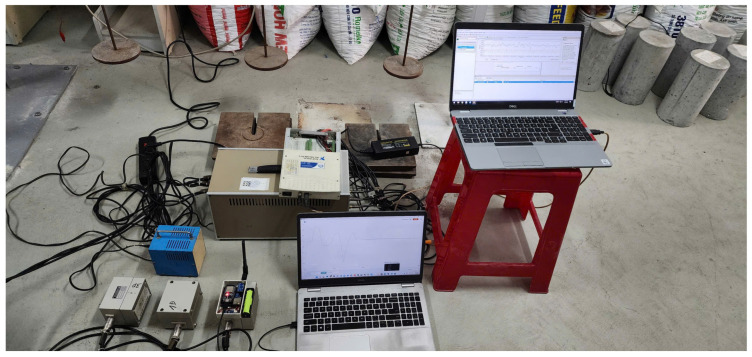
Laboratory validation setup of the proposed IoT sensing system and the commercial wired reference measurement system prior to bridge installation, enabling parallel signal acquisition and real-time data visualization.

Figure 14 shows the experimental setup of the wired reference system and the proposed IoT sensing system operating simultaneously during laboratory testing. Both systems were connected and configured to allow real-time viewing of vibration signals, enabling direct observation of signal behavior during excitation events. This parallel configuration establishes the basis for the subsequent comparison of measured acceleration responses, which is presented in the following subsection.

### 4.8. Data Visualization and Monitoring

**Figure 15:**
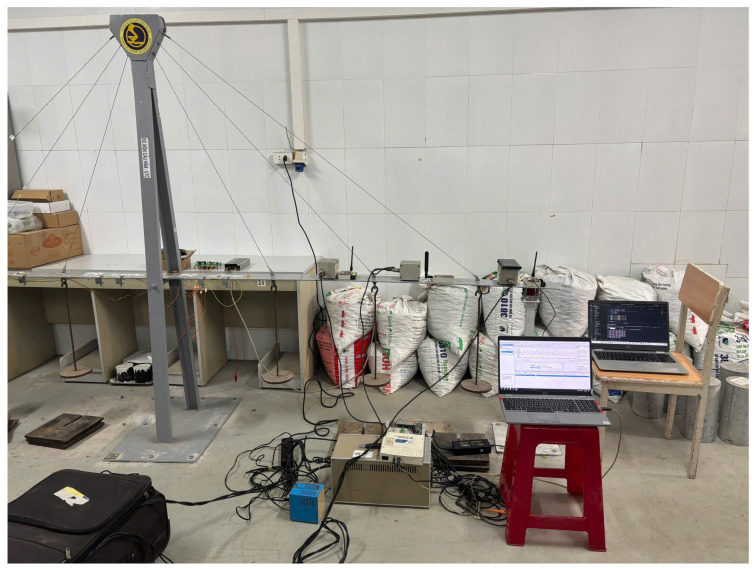
Laboratory-scale experimental setup for validation of the proposed SHM-IoT system, including distributed LoRa-based IMU sensor nodes, TDMA communication, and ESP32 gateway for real-time monitoring.

To evaluate the quality of the acquired vibration data and verify correct system operation, experiments are conducted on a laboratory-scale bridge model instrumented with multiple sensing nodes, as illustrated in Figure 15. The bridge model consists of a scaled deck structure supported by a tower and tensioned cables, representing a simplified cable-stayed bridge configuration.

Three LoRa-based IMU sensor nodes are deployed along the bridge deck at distinct locations, including positions near the left span, central region, and right span of the structure. This spatial distribution enables the system to capture variations in vibration response across different structural segments, particularly between regions close to supports and mid-span areas where dynamic responses are typically more pronounced.

During the experiment, all sensor nodes operate under a synchronized TDMA communication scheme and transmit vibration data simultaneously to a central ESP32-based gateway. The gateway forwards the collected data to monitoring laptops, where real-time visualization and logging are performed through a web-based dashboard. In parallel, selected measurements are compared with a reference measurement device integrated into the laboratory setup to verify the accuracy and consistency of the acquired data.

Based on this setup, the evaluation is conducted through two test scenarios. The first scenario focuses on individual sensor node validation, assessing the correctness and stability of measurements from a single node under controlled excitation. The second scenario evaluates the complete system under simultaneous multi-node operation, emphasizing synchronized data transmission, real-time visualization, and reliable data storage across all sensing nodes.

#### 4.8.1. Individual Sensor Node Validation

**Figure 16:**
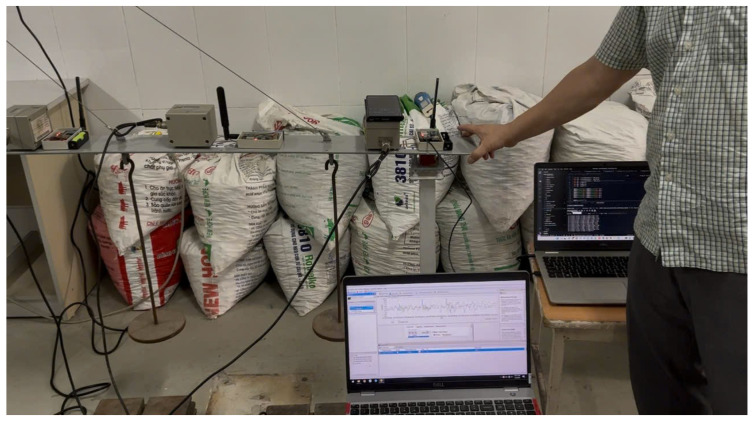
Inducing artificial vibrations in the right-side node of the structure and recording data through a wired connection to a computer.

The response of a single sensing node deployed at the right span of the laboratory bridge model is presented in this subsection. Figure 16 illustrates the experimental condition during which controlled vibration was applied locally to the structure while the sensor node remained fixed at the right-span location. During this test, acceleration and gyroscope signals were continuously measured and logged in real time through a wired connection to the monitoring computer.

**Figure 17:**
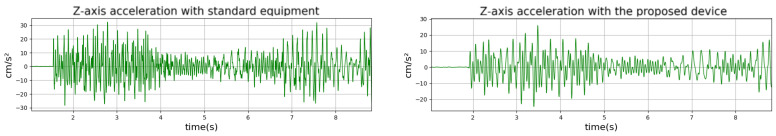
Time-domain comparison of Z-axis acceleration measured at the right span: (**left**) reference single-axis measurement device; (**right**) proposed IMU sensing node.

As the reference measurement device used in this experiment provides single-axis acceleration measurements only, validation is performed along a consistent measurement direction. Accordingly, the acceleration component along the Z-axis of the proposed sensing node is selected as the primary indicator for comparison. This axis corresponds to the dominant vertical response of the structure at the right-span location under the applied excitation. Figure 17 presents the time-domain Z-axis acceleration responses measured by the proposed sensing node and the reference measurement device. Both signals exhibit oscillatory behavior centered around zero, which reflects the dynamic nature of the induced vibration rather than static deformation. The amplitude of the oscillation increases during excitation intervals and decays toward zero as the vibration diminishes, indicating a stable dynamic response capture without observable offset or drift.

It should be noted that the proposed MPU6050-based sensing node operates at a sampling frequency of 200 Hz, whereas the commercial reference vibration measurement system acquires data at 1000 Hz. The higher sampling rate of the reference device is used for benchmarking purposes, while 200 Hz is sufficient to capture the dominant vibration characteristics of the laboratory-scale bridge model. The comparison presented in Figure 17 focuses on the consistency of structural vibration responses rather than sample-by-sample equivalence. The total observation window spans approximately 8 s (from ∼1 s to ∼9 s), with the primary excitation occurring between approximately 2 s and 5 s.

As observed in the upper plot, the commercial wired reference measurement device records peak acceleration values reaching approximately ±30–35 cm/s^2^, with dominant oscillations primarily distributed within the range of ±10–25 cm/s^2^ during the active excitation phase.

In contrast, the proposed sensing node exhibits peak acceleration values reaching approximately ±20–25 cm/s^2^, while the majority of oscillations are concentrated within approximately ±5–15 cm/s^2^. Compared to the reference system, the proposed device shows comparable excitation timing but slightly lower peak amplitudes, accompanied by smoother instantaneous extrema.

Despite differences in absolute peak values, both signals demonstrate highly consistent temporal behavior. The onset of vibration is observed at approximately t∼2 s, peak activity occurs during the interval from about 2.5 s to 4.5 s, and the vibration response gradually decays toward baseline after approximately 5–6 s. In both datasets, the decay phase exhibits a similar exponential-like attenuation trend, with acceleration amplitudes reducing to below approximately ±3–5 cm/s^2^ toward the end of the observation window.

Both signals remain centered around 0 cm/s^2^, with negligible DC offset (estimated mean value |μ|<2–3 cm/s^2^), confirming the absence of significant bias or long-term drift. Furthermore, the zero-crossing density and the apparent oscillation frequencies are visually consistent between the two signals, indicating that the dominant vibration frequency content is preserved.

The experimental comparison reveals a noticeable amplitude discrepancy between the MPU6050-based sensing node and the professional reference instrument. The available experimental data do not permit attribution of this discrepancy to a single dominant source. However, the sensing characteristics of the MPU6050, including measurement noise, bias instability, temperature sensitivity, limited resolution, and manufacturing tolerances, may contribute to differences in measured vibration amplitudes when compared with professional-grade accelerometers. Additional factors such as mounting conditions, sampling synchronization, internal filtering, and signal-conditioning procedures may also influence the observed responses.

It is important to note that the objective of this work is not to develop a metrology-grade vibration measurement instrument or to achieve reference-level measurement accuracy. Instead, the primary objective is to validate the feasibility and end-to-end operation of a low-cost distributed SHM-IoT architecture incorporating sensing, communication, synchronization, gateway integration, and cloud-based monitoring functions. Within this scope, the MPU6050 was selected because it provides a practical trade-off between cost, power consumption, integration complexity, and sensing capability.

Nevertheless, the observed amplitude differences indicate that the MPU6050 may not provide the level of accuracy required for applications involving precise modal identification, quantitative structural assessment, or long-term condition monitoring without additional calibration. Future work will therefore investigate sensor calibration procedures, temperature-compensation strategies, and the integration of higher-performance industrial-grade accelerometers to improve measurement accuracy while preserving the distributed architecture proposed in this study.

Nevertheless, the strong similarity in waveform envelope, excitation timing, and decay behavior indicates a high degree of correlation in the captured dynamic response. Qualitatively, the waveform correspondence suggests a strong temporal correlation (R>∼0.8, indicating strong qualitative temporal correlation between the two signals).

Overall, the results demonstrate that, under its 200 Hz sampling configuration, the proposed LoRa-based sensing node is capable of reliably capturing the dominant structural vibration responses, while the 1000 Hz reference signal serves as a benchmark for comparison.

- Temporal resolution consistent with the proposed 200 Hz node sampling configuration;
- Consistent waveform morphology and excitation timing;
- Comparable, though sensor-dependent, amplitude representation for dominant vibration components.

These observations confirm that the proposed sensing system can effectively capture key vibration characteristics relevant to structural health monitoring, particularly for applications focused on relative vibration trends, dynamic response tracking, and event detection, despite inherent differences in sensor technology.

#### 4.8.2. Frequency-Domain Evaluation of the Proposed Sensing Unit

In addition to the time-domain validation, a frequency-domain evaluation was performed to verify whether the proposed sensing unit can capture the dominant dynamic characteristics of the laboratory-scale bridge model. The Z-axis acceleration signal acquired by the MPU6050-based sensing node was processed using Welch’s power spectral density (PSD) method, which provides a robust estimate of the signal energy distribution over frequency by averaging windowed spectral estimates.

**Figure 18:**
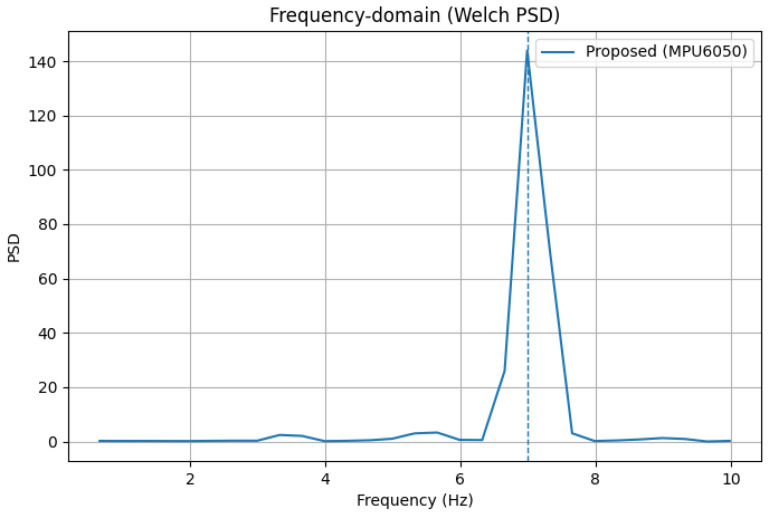
Frequency-domain evaluation of the proposed MPU6050-based sensing node using Welch’s power spectral density analysis of the Z-axis acceleration signal.

As shown in Figure 18, the PSD spectrum exhibits a clear dominant peak at approximately 7.1 Hz. This peak represents the fundamental vibration component captured by the proposed sensing unit during the bridge-model experiment. The identified frequency is consistent with previously reported experimental results for a comparable laboratory-scale cable-stayed bridge model, where accelerometer-based measurements identified the first vibration mode at approximately 7.0 Hz and fiber Bragg grating measurements reported a corresponding value of 7.118 Hz [23].

This result indicates that the proposed low-cost sensing node is capable of capturing the primary frequency component of the monitored structure, complementing the time-domain comparisons presented above. Nevertheless, comprehensive frequency-domain characterization, including higher-order mode identification, modal parameter estimation, frequency-response analysis, and long-duration operational modal evaluation, is beyond the scope of the present work. These aspects will be investigated in future studies after additional calibration and extended experimental validation of the sensing platform.

#### 4.8.3. Multi-Node Transmission and Data Storage

To evaluate the system-level performance under multi-node operation, three LoRa-based sensor nodes were activated simultaneously and assigned fixed transmission slots using a time division multiple access (TDMA) scheme. This configuration was designed to verify reliable concurrent data acquisition, wireless transmission, and backend storage without packet collision or data loss.

The three-node configuration was selected as a representative multi-node scenario for validating synchronization, TDMA slot allocation, collision-free communication, and end-to-end system integration. While larger deployments are possible according to the analytical TDMA capacity evaluation presented in Section 3, the objective of the present experiments is to verify the practical operation of the integrated architecture using real hardware rather than to benchmark the maximum network scale.

**Figure 19:**
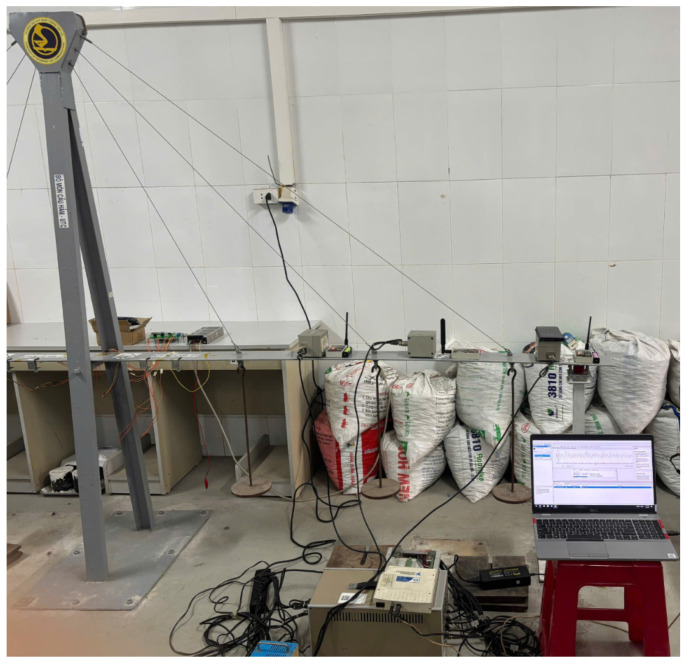
Deployment of three sensor nodes on the bridge model during multi-node operation.

The three sensor nodes were physically deployed at different spatial locations on the laboratory bridge model, corresponding to the left span, center span, and right span, as illustrated in Figure 19. All nodes were configured to continuously sense tri-axial acceleration data and transmit the measurements according to the predefined TDMA schedule. Status indicators on each node confirmed active sensing and wireless communication throughout the experiment.

**Figure 20:**
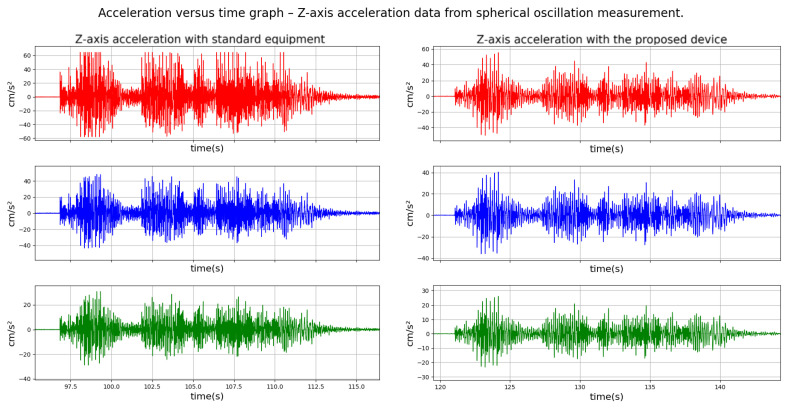
Time-domain comparison of Z-axis acceleration signals measured at three spatial locations along the bridge: (**top**) right span, (**middle**) center span, and (**bottom**) left span.

Figure 20 shows the experimental outcome of the multi-node test. The three Z-axis acceleration traces exhibit consistent vibration events at the deployed locations, while location-dependent amplitude differences reflect the spatial response of the bridge model.

**Figure 21:**
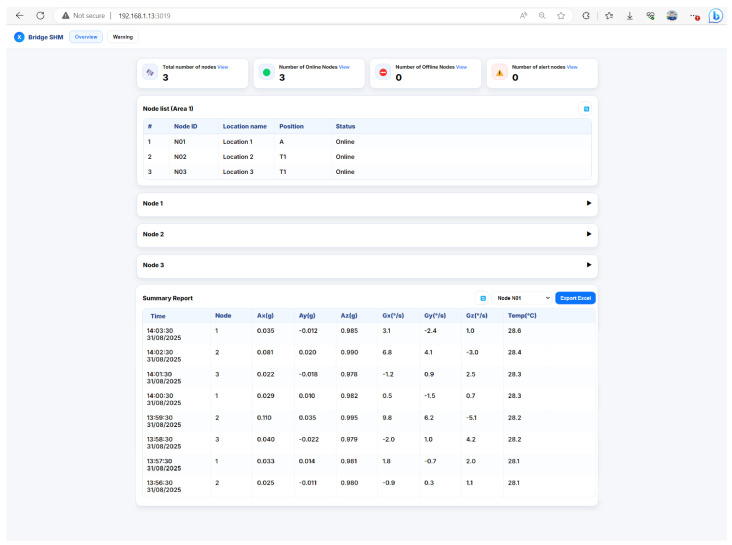
Real-time dashboard showing logged data from three sensor nodes received at the master gateway.

During system operation, all incoming data streams were visualized in real time using a graphical dashboard running on the monitoring computer, as shown in Figure 21. The dashboard concurrently displays acceleration data from all sensor nodes and continuously updates without interruption, demonstrating stable gateway operation and reliable data forwarding via the MQTT communication protocol.

**Figure 22:**
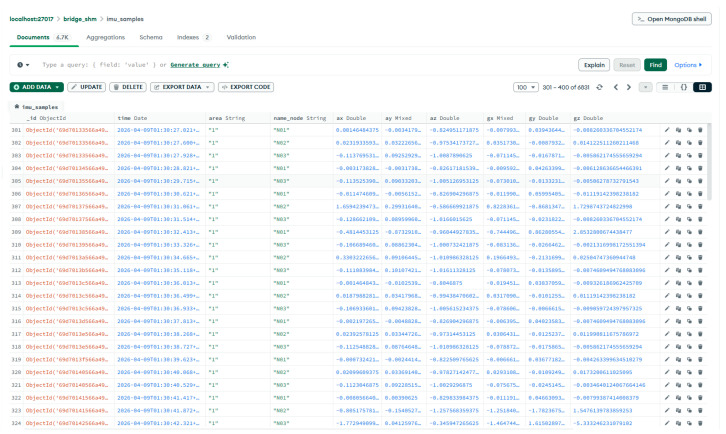
Database view showing stored data from three sensor nodes with ordered timestamps and node IDs.

The database snapshot in Figure 22 is included only to verify the experimental outcome: records from all three nodes were received, indexed by node ID, and stored with timestamps during the multi-node test.

These results confirm complete data reception, correct node-to-record association, and stable real-time visualization/storage during simultaneous multi-node operation.

In contrast to conventional wired measurement systems that require temporary deployment and manual data collection, the proposed architecture enables continuous, real-time monitoring through wireless communication and IoT integration. This supports long-term operation under real structural conditions without repeated setup.

Overall, the results confirm that the proposed system provides a reliable multi-node solution for distributed structural health monitoring, achieving a practical balance between measurement fidelity, system complexity, and deployment flexibility [24,25,26]. Scalability beyond the tested deployment is interpreted as an analytically evaluated architectural potential rather than an experimentally demonstrated result.

## 5. Discussion

The experimental results demonstrate that the proposed TDMA-based LoRa IoT architecture provides a reliable soft real-time communication framework for multi-node bridge structural health monitoring (SHM) systems. In contrast to conventional LoRaWAN-based sensing systems that rely on contention-based medium access mechanisms such as ALOHA or CSMA, the proposed approach introduces a time-division multiple access (TDMA) scheduling scheme designed according to the physical-layer characteristics of LoRa communication. This design enables predictable transmission timing, reduces packet collisions, and significantly improves communication reliability in the evaluated multi-node sensing environment.

One of the key findings of this study is the high communication reliability achieved by the proposed architecture. Experimental validation with multiple sensor nodes indicates that the system maintains a packet delivery ratio (PDR) exceeding 95% during continuous monitoring operations. This level of reliability is particularly important for SHM applications, where missing or delayed sensor measurements may affect the accuracy of the structural condition assessment and vibration analysis. By assigning dedicated transmission slots to each sensing node, the TDMA framework eliminates contention among nodes and ensures deterministic data transmission within the network.

Another important observation concerns the predictability of communication latency. The proposed TDMA communication framework operates with time slots ranging from approximately 100 ms to 200 ms depending on the configured LoRa parameters and the time-on-air (ToA) of transmitted packets. This predictable scheduling mechanism allows the sensing system to maintain stable data acquisition across multiple nodes without the latency fluctuations commonly observed in random-access communication schemes. As a result, the proposed architecture provides a deterministic sensing pipeline suitable for time-sensitive monitoring tasks such as vibration measurement and dynamic response analysis in bridge SHM systems.

The integration of a real-time operating system also plays a critical role in achieving stable system-level operation. In this work, FreeRTOS is not merely used as a firmware layer but is incorporated as a key architectural component that manages task scheduling, sensor data acquisition, LoRa communication, and network transmission processes. By distributing these tasks across multiple real-time threads and utilizing the dual-core architecture of the ESP32-WROOM-32D platform, the system effectively separates communication tasks from sensing and scheduling operations. This design improves system responsiveness and ensures that TDMA scheduling deadlines are maintained during continuous monitoring operation.

From a hardware perspective, the ESP32-WROOM-32D platform provides an efficient and cost-effective solution for implementing distributed sensing nodes in SHM applications. The dual-core microcontroller architecture allows parallel execution of networking and sensing tasks, while the integrated wireless capabilities simplify system integration and reduce hardware complexity. Compared with traditional embedded sensing platforms that often require separate microcontrollers for communication and sensing control, the ESP32-based design enables a compact and energy-efficient sensing node suitable for analytically planned multi-node deployment; however, large-scale physical deployment was not experimentally evaluated in this study.

In addition to the embedded sensing architecture, the proposed system integrates a backend monitoring platform that supports real-time data aggregation, storage, and visualization. The gateway collects sensor measurements from distributed LoRa nodes and streams the data to a cloud-based database using the MQTT protocol. The monitoring platform then provides a web-based dashboard for remote visualization of vibration signals and system status. This end-to-end architecture demonstrates the feasibility of integrating edge sensing, long-range wireless communication, and cloud-based monitoring into a unified SHM framework.

Table 14 compares the proposed architecture with representative LoRa-based SHM systems reported in recent literature. When compared with existing LoRa-based sensing systems reported in the literature, the proposed architecture offers several advantages. Many previously reported IoT monitoring systems rely on LoRaWAN infrastructure and random-access communication protocols, which may suffer from packet collisions and unpredictable latency when the number of sensor nodes increases. In contrast, the TDMA-based communication framework proposed in this study provides a deterministic slot-based framework for multi-node deployments by allocating transmission slots to each sensing node. Its scalability potential can be estimated analytically from the TDMA timing model, but larger physical deployments were not evaluated in the present study. This scheduling strategy improves communication stability and enables synchronized sensing operations across distributed nodes.

Furthermore, the PHY-aware TDMA scheduling mechanism proposed in this work considers the time-on-air characteristics of LoRa communication when defining slot durations and beacon synchronization intervals. By aligning the TDMA frame structure with the physical transmission properties of LoRa packets, the proposed system achieves efficient channel utilization while maintaining reliable communication performance. This approach is particularly beneficial for long-range sensing applications where communication bandwidth is limited and efficient medium access control is essential.

Overall, the experimental results confirm that the proposed TDMA-enabled LoRa IoT architecture provides a practical multi-node solution for bridge structural health monitoring applications. The combination of TDMA-scheduled communication, FreeRTOS-based embedded control, and an integrated backend monitoring platform enables reliable sensing with predictable data transmission behavior in the tested deployment. Claims regarding larger-scale operation are limited to analytical scalability potential based on the TDMA capacity model and are not presented as experimental validation. These characteristics are essential for long-term infrastructure monitoring systems, where stable communication performance and reliable data acquisition are critical for accurate structural condition assessment.

### 5.1. Limitations and Future Work

Despite the promising performance of the proposed TDMA-based LoRa IoT architecture, several limitations should be acknowledged. First, the experimental validation used three physical sensing nodes, which is sufficient for verifying end-to-end integration, synchronization, TDMA slot allocation, gateway forwarding, database storage, and real-time visualization, but it does not establish the maximum achievable network size. Scalability is therefore supported in this manuscript by the analytical TDMA capacity model rather than by large-scale physical deployment. Second, increasing the number of nodes primarily increases TDMA frame duration, network throughput demand, and end-to-end latency, whereas the local FreeRTOS task execution time on each sensor node remains governed by the same local task set. Therefore, network scalability and local FreeRTOS timing determinism are related but not identical issues. Third, synchronization accuracy distribution analysis and packet delivery characterization under varying communication distances were not experimentally performed in the present study. Future work will evaluate larger node counts, synchronization-error histograms or cumulative distributions, packet loss under distance variation, adaptive slot allocation, and long-term field deployment on operational bridges.

From a security perspective, the present study focuses on validating the proposed TDMA-LoRa communication architecture and lightweight security mechanisms under resource-constrained conditions. Comprehensive key lifecycle management, including secure key provisioning, secure key storage, periodic key rotation, and key compromise recovery procedures, was not implemented in the current prototype. These capabilities are important for operational deployments and represent a relevant direction for future development.

The cryptographic functions were implemented as a dedicated medium-priority FreeRTOS task, allowing TDMA synchronization and packet scheduling tasks to maintain their timing behavior independently of security processing. The present study focused on validating communication reliability, synchronization accuracy, security functionality, and structural vibration monitoring performance within the proposed TDMA-LoRa SHM architecture. Although the implemented cryptographic mechanisms were executed as an independent FreeRTOS task to avoid interference with TDMA slot scheduling, a dedicated evaluation of computational overhead, timing overhead, memory footprint, and energy consumption attributable solely to the security functions was not performed.

Future work will therefore include a comprehensive characterization of cryptographic overhead, including processor utilization, execution latency, memory requirements, and energy consumption under different security configurations. Such analysis will provide a more detailed assessment of the trade-offs between security strength and system resource utilization in large-scale SHM deployments.

Although battery-powered sensor nodes were used during experimental validation to simplify installation and avoid additional wiring, the primary objective of this study was to evaluate communication reliability, synchronization performance, and vibration monitoring capability in the proposed TDMA-LoRa SHM system. In practical SHM deployments, sensor nodes may be powered directly from available infrastructure power sources, reducing the importance of battery lifetime considerations.

Consequently, detailed power-consumption profiling and state-dependent power-consumption curves were not included in the present study. Nevertheless, energy-efficient operation remains an important consideration for future battery-powered or hybrid solar-assisted deployments. Future work will therefore investigate power consumption across different operating states, duty-cycle optimization strategies, and long-term battery lifetime evaluation under various deployment conditions.

### 5.2. Scalability Considerations for Large-Scale Deployments

Only three physical sensor nodes were experimentally validated in this study; therefore, large-scale bridge deployments involving dozens or hundreds of nodes were not tested. The current implementation employs a single-channel TDMA LoRa schedule, in which each sensor node is assigned one dedicated uplink slot within a beacon-synchronized frame. As the number of nodes increases, the number of required slots increases approximately linearly, causing the TDMA frame duration, per-node update interval, and end-to-end network latency to increase. This limitation originates primarily from channel occupancy and LoRa packet airtime rather than from computational limitations of the ESP32 platform. The local FreeRTOS scheduling behavior of each sensor node is largely independent of the total number of nodes participating in the network because each node executes an identical local task set regardless of deployment size. Increasing network size therefore primarily affects communication scheduling rather than local task execution timing.

For this reason, the proposed architecture should be regarded as directly suitable for small- to medium-scale SHM deployments under the current single-channel implementation. Adaptation to larger bridge deployments would require additional network-level scaling mechanisms. The architectural extension path consists of: (1) multi-channel LoRa operation, where sensor nodes are distributed across multiple frequency channels to reduce the number of slots per channel; (2) hierarchical TDMA scheduling, where local cluster coordinators manage groups of nearby nodes and forward aggregated data to the gateway; (3) gateway clustering and spatial network segmentation, where long bridge structures are divided into communication zones served by different gateways; and (4) hybrid scheduling approaches that combine TDMA with channel diversity to preserve deterministic access while reducing frame length. These approaches are discussed as future architectural extensions only and have not been implemented or experimentally validated in the present work. The manuscript therefore avoids presenting the current prototype as a demonstrated large-scale network and limits scalability claims to the analytical TDMA capacity model and the proposed architecture path for future deployment expansion.

Future work will also investigate the integration of AI-based vibration pattern analysis for early damage detection in bridge structures.

## 6. Conclusions

This paper presented the design and experimental validation of a soft real-time IoT architecture for bridge Structural Health Monitoring (SHM) based on LoRa communication and a TDMA scheduling mechanism implemented on an embedded FreeRTOS platform. The proposed system integrates distributed vibration sensing nodes, a LoRa-based TDMA-scheduled communication layer, an embedded gateway, and a web-based monitoring platform as an architecture designed for multi-node SHM deployments. The experimental validation focuses on representative end-to-end operation rather than maximum-scale network benchmarking.

A key aspect of the system design is the selection of the ESP32-WROOM-32D module as the hardware platform for both sensing nodes and the gateway. The dual-core architecture of the ESP32 enables efficient task partitioning between network communication and time-critical sensing operations. In the proposed implementation, WiFi networking, TCP/IP processing, and MQTT data transmission are executed on one processor core, while LoRa communication, TDMA slot scheduling, and sensor data acquisition are handled on the second core. This architecture ensures stable real-time execution and reduces timing interference between concurrent tasks in multi-node sensing environments.

The synchronization experiments presented in this study evaluate short-term laboratory operation and do not provide a long-term characterization of clock drift. In practical bridge SHM deployments, oscillator frequency variations caused by temperature changes, component aging, and hardware tolerances may affect synchronization accuracy over extended periods of operation. To reduce drift accumulation, the proposed TDMA framework employs periodic gateway beacon synchronization at the beginning of each TDMA frame, allowing sensor nodes to regularly update their local timing reference after beacon reception. Furthermore, guard intervals are incorporated into the TDMA schedule to accommodate residual synchronization uncertainty, oscillator drift, communication latency variations, and software scheduling jitter. However, the effectiveness of this approach under long-duration operation and varying environmental conditions, including temperature-induced oscillator drift of ESP32-based sensor nodes, has not been experimentally validated in the present work. Comprehensive evaluation of long-term synchronization stability, temperature-dependent oscillator behavior, and clock-drift accumulation in real bridge environments remains an important direction for future research.

Another important contribution of this work lies in the system-level integration of FreeRTOS within the communication architecture. Rather than serving solely as a firmware abstraction layer, FreeRTOS is used as a core system management component that coordinates sensing, communication, and networking processes across multiple tasks. This real-time task scheduling capability enables accurate execution of TDMA transmission cycles and supports deterministic multi-node communication in the LoRa network.

Experimental validation demonstrates that the proposed TDMA-based LoRa communication framework achieves reliable multi-node data transmission with a packet delivery ratio exceeding 95% in the three-node laboratory deployment. The implemented TDMA scheduling mechanism supports transmission slots in the range of approximately 100–200 ms, allowing the tested sensing nodes to transmit vibration measurements within a predictable communication cycle. Timing analysis further confirms that the real-time scheduling framework maintains highly stable slot synchronization with microsecond-level timing errors and negligible jitter relative to the TDMA slot duration. These results validate deterministic operation for the tested deployment, while larger-scale scalability remains supported analytically and requires future experimental verification.

The experimental results also verify the stability of the end-to-end monitoring pipeline, including sensor data acquisition, wireless transmission, gateway aggregation, MQTT data streaming, and web-based visualization. The integrated monitoring system enables continuous collection and remote analysis of structural vibration data, providing a practical solution for long-term infrastructure monitoring.

Overall, the proposed architecture demonstrates that combining TDMA-based LoRa communication with a FreeRTOS-driven embedded platform can provide deterministic and reliable wireless monitoring for multi-node bridge SHM applications. The presented system offers a cost-effective framework whose scalability potential is described by the TDMA capacity model, but larger physical deployments and distance-dependent packet-loss characterization are left for future validation.

## Figures and Tables

**Table 1:** Comparison of representative SHM networking systems in terms of communication protocol, real-time execution support, synchronization mechanism, latency characteristics, scalability, and communication range.

System	Communication Protocol	RTOS Support	Time Synchronization	Data Latency	Scalability	Communication Range
Proposed system (this work)	LoRa (Custom TDMA)	Lightweight RTOS/Em- bedded scheduler	Gateway-based slot syn- chronization	Deterministic, bounded by TDMA slot; E2E latency depends on pipeline	Controlled via TDMA slots	Long-range (km-level)
LoRaWAN-based SHM systems	LoRaWAN (ALOHA)	Typically bare-metal or lightweight OS	Class A: asynchronous uplink; downlink only in RX windows. Class B: synchronized via network beacon	Unpredictable (random access) due to ALOHA collisions	Limited due to collisions	Long-range (km-level)
ZigBee SHM (IEEE 802.15.4)	IEEE 802.15.4 (CSMA/CA)	TinyOS/Contiki (commonly used)	Beacon-enabled superframe synchronization; GTS provides guaranteed slots	Moderate and contention-based	Limited by mesh overhead	Short-range (10–100 m)

**Table 2:** The data field, after being decoded and stored in the database, has an identifier and can be accessed via timestamp.

Field	Type	Description
time	Date	Server-side timestamp of data reception
area	String	Identifier for the monitoring area (default ‘1’)
name_node	String	Unique identifier for the sensor node (e.g., ‘N01’)
ax	Number	Acceleration data (x-axis)
ay	Number	Acceleration data (y-axis)
az	Number	Acceleration data (z-axis)
gx	Number	Gyroscope angular velocity data (x-axis)
gy	Number	Gyroscope angular velocity data (y-axis)
gz	Number	Gyroscope angular velocity data (z-axis)
temp	Number	Temperature data

**Table 3:** Time-on-Air and TDMA slot length for different LoRa PHY configurations.

SF	BW (kHz)	Payload (B)	ToA (ms)	Slot Length (ms)	Max Nodes per Frame
7	125	37	82.4	90	11
8	125	37	148.5	160	6
9	125	37	264.2	280	3
10	125	37	494.6	520	1

**Table 4:** Secure TDMABeacon format (16-byte Ciphertext over LoRa).

Field	Size	Description
Beacon Data	12 bytes	TDMA synchronization information
MIC	4 bytes	Message authentication code

**Table 5:** Unified RTOS task model for gateway and sensor nodes.

Task	Function	Period	Priority	Role
IMU Sampling Task	Acquire vibration data from the IMU sensor	5 ms	Medium	Sensor Node
TDMA Scheduler Task	Manage TDMA frame timing, process synchronization beacons, compute slot start time	Frame-based	High	Sensor Node
LoRa TX Task	Transmit encrypted packets during assigned TDMA slot	Slot-based	High	Sensor Node
LoRa RX Task	Receive radio packets; on nodes: receive sync beacons; on gateway: receive uplink packets	Event-driven	High	Both
Encryption Task	Perform AES encryption and generate message integrity code (MIC) before wireless transmission	On-demand	Medium	Sensor Node
Beacon Generation Task	Compose and broadcast synchronization beacons to define TDMA frame epoch	Per-frame	High	Gateway
MQTT Publishing Task	Push received ciphertext packets with metadata to backend server via MQTT	Asynchronous	Low/Medium	Gateway

**Table 6:** Statistical timing characteristics of gateway FreeRTOS tasks (TDMA Scheduler, LoRa RX, MQTT Publisher).

Task	Samples	Avg (μs)	WCET (μs)	BCET (μs)	Jitter (μs)	StdDev (μs)	95% CI of Avg (μs)
TDMA	1000	4631.212	5046	3446	1600	402.83	±24.97
LORA_RX	1000	951.96	1019	722	297	49.20	±3.05
MQTT Publisher	1000	5341.059	8669	2268	6401	1165.03	±72.21

**Table 7:** Statistical timing characteristics of sensor node FreeRTOS tasks (IMU sampling, lora processing, encryption task).

Task	Samples	Avg (μs)	WCET (μs)	BCET (μs)	Jitter (μs)	StdDev (μs)	95% CI of Avg (μs)
IMU Sampling	1000	2668.434	3623	2649	974	31.829	±1.97
LORA Processing	1000	2720.267	3011	2648	363	85.279	±5.29
Encryption	1000	102.307	443	99	344	10.791	±0.67

**Table 8:** Timing error and jitter metrics for TDMA slot execution.

Mean Error (μs)	Max Deviation (μs)	Min Deviation (μs)	Jitter (μs)	StdDev (μs)	95% CI of Mean (μs)
3.04	11	3	8	0.6	±0.04

**Table 9:** End-to-end latency breakdown of the proposed SHM–IoT data pipeline.

Pipeline Stage	Avg Latency (ms)
Sensor Acquisition → Encryption	0.024–0.044
Encryption → LoRa TX Start	0.89–10.890
Sensor Node Processing Total	0.89–10.934
LoRa Transmission (Time-on-Air)	∼345
Gateway Processing	25.33
MQTT Transport	–
Database Commit	∼1.8
Total End-to-End Latency	∼390–410

**Table 10:** Packet Delivery Ratio (PDR) for each sensor node.

Node ID	Packets Received	Packets Lost	Total	PDR (%)
1	1106	35	1141	96.93%
2	1130	15	1145	98.69%
3	1122	14	1136	98.77%

**Table 11:** Comparison of sensor node latency at each processing step.

Processing Component	Non-RTOS	RTOS
Sensor Read	2670 µs	2668 µs
Encryption	109–383 µs	102–443 µs
LoRa TX (physical ToA)	459–460 ms	345 ms
Total Pipeline (Sensor → Encrypt → TX-ready)	459 ms	5.39–7.07 ms

**Table 12:** Comparison of timing and transmission parameters between RTOS and Non-RTOS systems.

Metric	Non-RTOS	FreeRTOS + TDMA
Loop Jitter	0.03 → 8.8 ms	1.6 ms (Equation (20))
Packet Delivery Ratio (multi-node)	frequently overlapping	96.9–98.8%

**Table 13:** Comparison between the commercial wired reference system and the proposed TDMA-based LoRa IoT sensing system.

Aspect	Commercial Wired Reference System	Proposed IoT-Based System
Measurement principle	Servo-type vibration meter (IMV VM-5112)	MEMS-based accelerometers
Sensor type	VP-5112 servo-type pickup	Embedded tri-axis IMU
Measurement axes	Single-axis (per pickup)	Tri-axis
Acceleration measurement range	0.1–1000 cm/s^2^	Up to ±15,700 cm/s^2^ (±16 g IMU range)
Measurable frequency range	DC—100 Hz	≈1–1100 Hz (system-limited)
Sensitivity/accuracy	Linearity error ±0.15% F.S.	Lower sensitivity, few %
Sampling capability	High, period setup	Moderate, period setup
Communication	Wired	Wireless (LoRa)
Installation	Manual, temporary	Permanent, distributed
Monitoring mode	On-site laboratory testing	Remote, real-time monitoring

**Table 14:** Compares the proposed architecture with representative LoRa-based SHM systems reported in recent literature.

Study	Communication	Nodes	PDR	Deterministic	SHM Application
Loubet et al., 2019 [27]	LoRaWAN (ALOHA)	Multiple	>90%	No	Construction SHM monitoring
Truong et al., 2020 [11]	LoRa WSN	3–5	Not reported	No	Bridge vibration monitoring
Park et al., 2023 [13]	LoRa LPWAN	Multi-node	∼92%	No	Large bridge SHM system
Ragnoli et al., 2022 [15]	LoRa WSN	Multiple	Not reported	No	Structural inclination monitoring
Rahita et al., 2024 [28]	IoT wireless sensing	Multi-node	Not reported	No	IoT-based infrastructure SHM
Qiao et al., 2025 [16]	LoRa synchronized nodes	2	>90%	Partially	Footbridge modal identification
Edge-SHM Architecture (MDPI Sensors) [29]	LoRa wireless sensing	Multi-node	∼90%	No	Edge-based vibration monitoring
Various LoRaWAN SHM systems [2]	LoRaWAN MAC	Multi-node	85–92%	No	Civil infrastructure monitoring
This work	TDMA-based LoRa	Multi-node	>95%	Yes	Bridge vibration SHM with TDMA-scheduled communication

## Data Availability

The data that support the findings of this study are available from the corresponding author upon reasonable request. Due to privacy and operational restrictions, the datasets are not publicly accessible, but may be shared for academic research purposes in compliance with institutional and ethical guidelines.

## Abbreviations

The following abbreviations are used in this manuscript:

AES	Advanced Encryption Standard
ALOHA	ALOHA Medium Access Protocol
BW	Bandwidth
CMAC	Cipher-based Message Authentication Code
CSMA	Carrier Sense Multiple Access
ECB	Electronic Codebook
IEEE	Institute of Electrical and Electronics Engineers
IMU	Inertial Measurement Unit
IP	Internet Protocol
IoT	Internet of Things
KENC	Encryption Key
KMIC	Message Integrity Key
LoRa	Long Range
LPWAN	Low Power Wide Area Network
MCU	Microcontroller Unit
MIC	Message Integrity Code
MQTT	Message Queuing Telemetry Transport
PHY	Physical Layer
RTOS	Real-Time Operating System
SF	Spreading Factor
SHM	Structural Health Monitoring
TDMA	Time Division Multiple Access
